# PulseCam: a camera-based, motion-robust and highly sensitive blood perfusion imaging modality

**DOI:** 10.1038/s41598-020-61576-0

**Published:** 2020-03-16

**Authors:** Mayank Kumar, James W. Suliburk, Ashok Veeraraghavan, Ashutosh Sabharwal

**Affiliations:** 1 Electrical and Computer Engineering, Rice University, 6100 Main St, Houston, TX 77005 USA; 2 Division of General Surgery, Baylor College of Medicine, 6620 Main St, Houston, TX 77030 USA

**Keywords:** Diagnostic markers, Electrical and electronic engineering, Imaging and sensing

## Abstract

Blood carries oxygen and nutrients to the trillions of cells in our body to sustain vital life processes. Lack of blood perfusion can cause irreversible cell damage. Therefore, blood perfusion measurement has widespread clinical applications. In this paper, we develop PulseCam — a new camera-based, motion-robust, and highly sensitive blood perfusion imaging modality with 1 mm spatial resolution and 1 frame-per-second temporal resolution. Existing camera-only blood perfusion imaging modality suffers from two core challenges: (i) motion artifact, and (ii) small signal recovery in the presence of large surface reflection and measurement noise. PulseCam addresses these challenges by robustly combining the video recording from the camera with a pulse waveform measured using a conventional pulse oximeter to obtain reliable blood perfusion maps in the presence of motion artifacts and outliers in the video recordings. For video stabilization, we adopt a novel brightness-invariant optical flow algorithm that helps us reduce error in blood perfusion estimate below 10% in different motion scenarios compared to 20–30% error when using current approaches. PulseCam can detect subtle changes in blood perfusion below the skin with at least two times better sensitivity, three times better response time, and is significantly cheaper compared to infrared thermography. PulseCam can also detect venous or partial blood flow occlusion that is difficult to identify using existing modalities such as the perfusion index measured using a pulse oximeter. With the help of a pilot clinical study, we also demonstrate that PulseCam is robust and reliable in an operationally challenging surgery room setting. We anticipate that PulseCam will be used both at the bedside as well as a point-of-care blood perfusion imaging device to visualize and analyze blood perfusion in an easy-to-use and cost-effective manner.

## Introduction

Measuring peripheral blood perfusion, i.e. the flow of blood to end-organs and tissue through the blood vessels, has widespread clinical applications such as assessment of wounds and burns^1,2^, diagnosing peripheral arterial disease (especially in patients suffering from diabetes^2,3^), monitoring micro-circulation to identify shock in critical care^4–6^, and for monitoring blood flow at surgical sites, e.g. during plastic surgeries^7^ and surgical revascularization procedures^8^.

Popular clinical markers of local or peripheral perfusion such as center-to-toe temperature difference, skin mottling and capillary refill time are subjective indicators that lack the required sensitivity and specificity to identify patients with compromised peripheral perfusion^9^. In the last few decades, non-invasive contact-based optical techniques have increasingly been adopted in clinical settings for quantitative and qualitative assessment of blood perfusion in peripheral tissue. Specifically, Near-infrared Spectroscopy (NIRS) has been used to measure tissue oxygenation and hemoglobin concentration^10–12^, and a pulse oximeter (PulseOx) is routinely used to measure arterial oxygen saturation (SpO2)^13^. Advanced PulseOx can also measure peripheral perfusion index (PPI) that is used in both research and clinical settings as a non-invasive marker of peripheral perfusion^14–18^. Laser Doppler Flowmetry (LDF) is another popular modality used to measure micro-circulatory blood flow in tissue, especially to assess wound healing and for skin disease research^19^.

**Figure 1:**
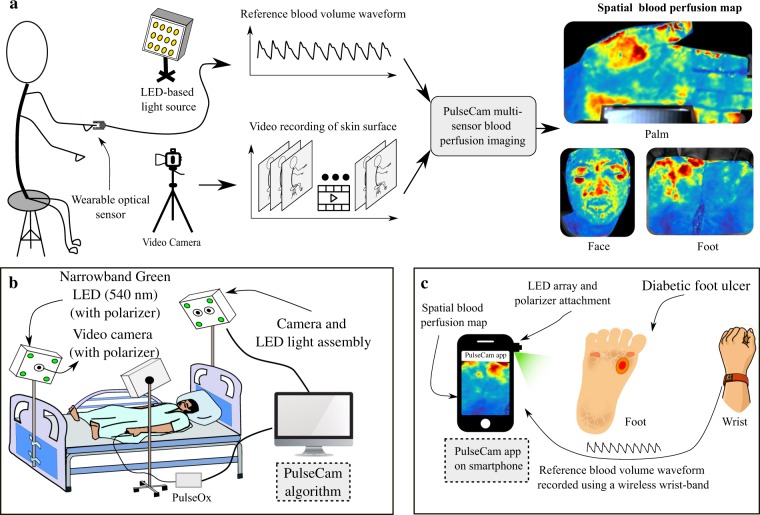
PulseCam — A camera-based multi-sensor blood perfusion imaging modality: (**a**) PulseCam combines the video recording of the skin surface (or internal tissue) and a reference blood volume waveform to reliably obtain blood perfusion map over the entire imaged skin surface or tissue region, (**b**) a schematic diagram of PulseCam used as a bedside perfusion imaging system in critical care and the operating room, (**c**) a schematic diagram of PulseCam used as a hand-held imaging tool to visualize blood perfusion at surgical sites, wounds and ulcers.

However, all of these contact-based optical modalities can only measure blood perfusion at a specific location on the skin surface, i.e., the point-of-contact, and their measurements are sensitive to the exact placement of the probe. The high spatial variability in blood perfusion across tissue such as the skin limits the clinical utility of such single point contact-based modalities^20^. In this paper, we develop a novel camera-based, multi-sensor, motion-robust blood perfusion imaging modality, named PulseCam, that can reliably measure spatial maps and temporal trends of peripheral blood perfusion over the skin surface or internal tissue. Figure 1(a) illustrates the various components of our new PulseCam blood perfusion imaging system. PulseCam can be implemented both as a bedside patient monitoring system, e.g., in an ICU or the operating room (see Fig. 1(b)), as well as a hand-held imaging tool to visualize blood perfusion at surgical sites, wounds, and ulcers (see Fig. 1(c)).

The volume of blood in the microvasculature just underneath the skin surfaces changes periodically due to the pulsatile beating of the heart. This pulsatile change in blood volume can be measured by recovering the associated change in light absorption by the hemoglobin in the blood — a technique known as photoplethysmography^21^. The amplitude of the pulsatile blood volume waveform is directly proportional to the amount of arterial blood reaching the imaged tissue, and therefore, can be used as a non-invasive, quantitative and a surrogate measure of peripheral blood perfusion^22^. Conventionally, a contact-based PulseOx is employed to measure this pulsatile amplitude at few peripheral locations directly, e.g., fingers, toe, forehead, etc., and is commonly known as peripheral perfusion index (PPI)^15^. More recently, several researchers have shown the feasibility of recovering the same blood volume waveform from only the video recording of the skin surface, and thereby, opened up the possibility of obtaining spatial maps of peripheral blood perfusion amplitude, more commonly known as imaging photoplethysmography or PPG imaging^23–25^, as well using a camera.

However, a non-contact camera-based system that operates from a distance records a significantly weaker blood volume waveform from any imaged skin region or internal tissue compared to a contact-based PulseOx. Therefore, prior^26–29^ camera-only blood pulsation imaging system usually suffers from the challenge of significantly smaller signal-to-noise ratio. The small blood volume waveform, i.e., the signal of interest, rides on top of a much larger (approx. 100×) reflection from the skin surface or the internal tissue that is not of any importance for perfusion imaging. Moreover, the surface reflection can change drastically with even a slight movement of the skin, making the small blood perfusion-related signal recovery even more difficult in the presence of motion. As a result, recent attempts to obtain spatial maps of pulsatile blood perfusion using camera-only approaches have resulted in noisy and motion-corrupted perfusion estimates^26–29^ with limited clinical value.

The possibility of obtaining the blood pulsation amplitude map from camera-based recording of tissue under ambient lighting was first demonstrated by^23^. They directly estimated the power of the recorded intensity variation signal at each pixel around the heart rate to obtain power map of pulsation. However, such power maps can easily be corrupted by motion artifacts and variations in light intensity across the skin surface as noted by the authors as well. Then^24^, proposed to compute a (complex) inner-product between a reference blood volume (or PPG) waveform and the waveform of the reflected light recorded by each pixel of the camera to obtain blood pulsation amplitude map. Since the reference PPG waveform is formed by averaging the reflected light from a large user-selected area of the same imaged skin surface, so it can also be corrupted by motion artifacts. Therefore, camera-only locked-in amplification also suffers from the challenge of correlated noise and motion artifacts, e.g., during motion both the reference pulse waveform and the waveform obtained at each camera pixel would be similarly corrupted. More recently^27^, used chrominance-based color channel mapping (CHROMO) approach to reduce impact of motion artifact on pulsatile amplitude estimation. However, they also used a reference pulse waveform from the same skin surface to obtain blood pulsation amplitude maps.

To avoid the challenges associated with camera-only blood pulsation imaging, in PulseCam, we propose to record a reliable reference blood volume waveform simultaneously using a contact-based wearable optical sensor placed at a convenient and well-perfused site on the skin surface such as the index finger or the toe or the forehead. The reference location can be chosen to be farther away from the investigated skin surface, and therefore, our proposed blood perfusion imaging modality is non-contact w.r.t. the skin or tissue surface under investigation. We recently^30^ showed the initial feasibility of such a multi-sensor approach to blood perfusion imaging by performing locked-in amplification of the video recording with the reference blood volume waveform obtained using the PulseOx to derive reliable blood perfusion maps in the palm during and after an arterial blood flow occlusion.

In this paper, we devised a completely new algorithm to combine the video recording of the skin or tissue surface under investigation with the reference blood volume waveform recorded simultaneously using the PulseOx. Instead of using the locked-in amplification, i.e, the normalized inner product, to estimate blood perfusion amplitude map, here we propose to use a robust M-estimators that automatically rejects statistical outliers in the video recording due to (i) sudden variation in surface reflections caused by motion artifacts, skin rotation and motion tracking errors, and (ii) camera measurement noise. Further, in this work, we propose to use a novel brightness invariant optical flow algorithm^31^ to reliably track and compensate the motion of the skin surface during perfusion imaging. Existing approaches to motion-robust blood perfusion imaging^25,27,32,33^ relies on standard optical flow algorithm^34,35^ for tracking the selected skin region before estimating the blood perfusion map. Since the appearance of the skin surface changes over time due to the blood volume change underneath, therefore standard optical flow algorithms that assume brightness constancy cannot be used for video stabilization. In this paper, we show, for the first time, that our novel approach of using a brightness invariant optical flow algorithm^31^ reduces the error in blood perfusion estimate below 10% in diverse motion scenarios compared to 20–30% error when using existing optical flow algorithms that assume brightness constancy as is usually done.

We evaluated PulseCam both in a lab-based study as well as in a clinical context. In the lab-based study, we conducted several controlled blood flow occlusion experiments on healthy participants of varying skin tones to quantify and compare the performance of PulseCam with a contact-based commercial PulseOx and an infrared thermal camera. Through these experiments, we demonstrate that PulseCam can easily detect arterial and venous blood flow occlusion and identify different levels of partial blood flow occlusion. In contrast, the perfusion index obtained using a PulseOx does not register an appreciable reduction in perfusion during partial or venous occlusion. Further, we found that the PulseCam has much higher spatial resolution and has at least two times better sensitivity and 2–3 times better response time in detecting blood flow occlusion compared to an infrared thermal camera. And, PulseCam can achieve all this at a much lower cost compared to an infrared thermal camera.

Next, we evaluate the robustness and effectiveness of PulseCam system in a clinical setting during a surgery in which general anesthesia is used. It is known that anesthesia induction results in a block of sympathetic tone that leads to a sudden drop in the peripheral sympathetic nerve activity. Reduction in peripheral nerve activity leads to vasodilation that causes an increase in micro-circulations. Recently^36^, also tested a camera-based blood perfusion imaging system to obtain changes in micro-circulation amplitude maps in the palm during regional anesthesia procedures. In our pilot clinical study conducted during different types of surgeries, we establish that PulseCam shows statistically significant (*p* < 0.05) change in micro-circulatory perfusion associated with anesthesia-induced vasodilation whereas perfusion index measured using a PulseOx does not, and therefore PulseCam is robust and reliable and can be translated as a blood perfusion imaging system in a challenging clinical setting.

## Results

To evaluate the performance of PulseCam, first we compare the perfusion estimate obtained using PulseCam against perfusion index obtained using a PulseOx as well as with the skin temperature map obtained using an infrared thermal camera in a lab-based study on healthy participants of varying skin tones. Next, in a clinical context, we compare PulseCam against a commercial PulseOx derived perfusion index in an operationally challenging setting to monitor changes in peripheral perfusion of patients during surgery performed under general anesthesia.

### Comparing PulseCam and PulseOx

To compare blood perfusion obtained using PulseCam and perfusion index measured using a PulseOx, we perform controlled brachial artery blood flow occlusion experiment by placing a manual pressure cuff around the upper arm of healthy participants to systematically decrease the blood perfusion in the palm region. The level of pressure applied is known as *Blood Occlusion Pressure* (BOP) and is measured in mmHg. Here, we perform two related studies to evaluate and compare perfusion estimates obtained from the camera-based PulseCam and contact-based PulseOx sensor: (i) arterial and venous occlusion study, and (ii) incremental vascular occlusion study. These studies were approved by Rice University IRB (Protocol number 928192-1), and informed consent was obtained from all the participants.

#### Arterial and venous occlusion study

**Figure 2:**
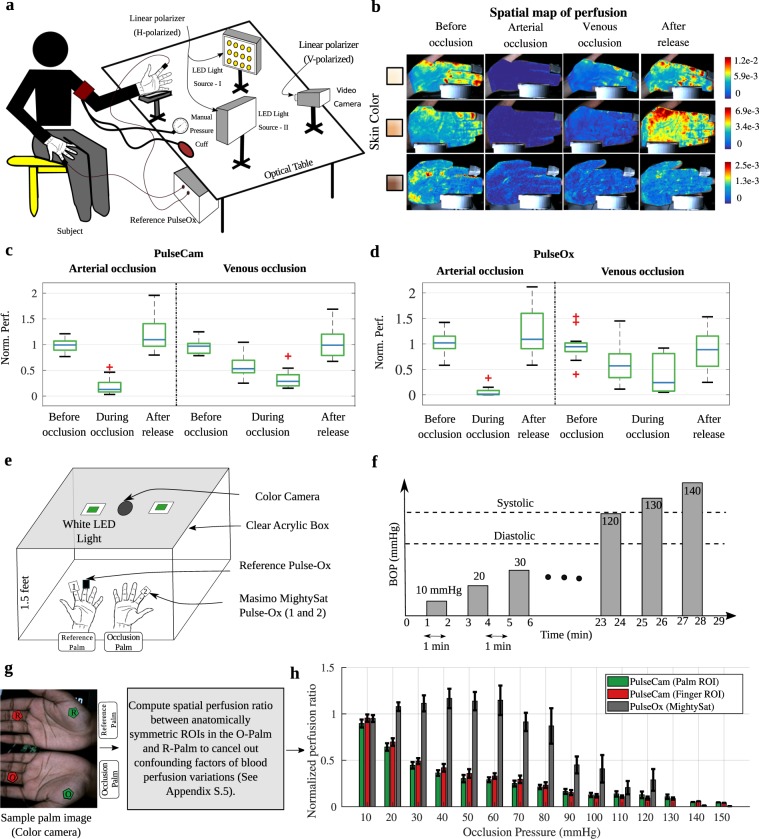
Comparing performance of PulseCam and PulseOx to measure perfusion during occlusion study: (**a**) Arterial and venous occlusion experimental setup, (**b**) Spatial map of blood perfusion during different stages of arterial and venous occlusion for 3 (out of 12) participants of varying skin tones, (**c,d**) Box plot of normalized perfusion obtained using PulseCam (**c**) and PulseOx (**d**) at different stages of occlusion for 12 participants of varying skin tones; PulseCam can be used to distinguish between arterial, venous and no-occlusion scenarios, whereas a PulseOx has difficulty detecting a venous occlusion, (**e**) Incremental vascular occlusion experimental setup, (**f**) Incremental vascular occlusion experimental protocol, (**g**) Normalized perfusion ratio measured using PulseCam (at the selected ROI in the palm and the finger) and MightySat PulseOx at different levels of occlusion for 15 participants of varying skin tones. PulseCam can easily detect perfusion reduction due to partial blood occlusion pressure between 10–70 mmHg whereas a PulseOx does not show any appreciable decrease in measured perfusion, and therefore cannot be used to detect partial or venous occlusion; Therefore, PulseCam has substantially higher sensitivity compared to PulseOx.

In the first study, we induce large blood perfusion variations in the palm of 12 healthy participants (5 female, 7 male) of varying skin tones (Fitzpatrick skin type I to VI) by either completely occluding the arterial inflow of blood (arterial or total occlusion, BOP ≈ 140 mmHg) or by only stopping the venous back-flow of blood (venous or partial occlusion, BOP ≈ 70 mmHg) using the manual pressure cuff. Figure 2(a) shows a schematic diagram of our experimental setup consisting of a CMOS color camera, a pulse oximeter, and two white LED light sources. We also place polarizer both on the camera as well as on the light source to minimize specular reflection. More details about the experimental setup, protocol, and the study participants can be found in Supplementary S.5.

Figure 2(b)  shows the spatial maps of blood perfusion in the palm region obtained using PulseCam at different stages of occlusion for 3 participants (out of 12) of widely varying skin tones. Clearly, PulseCam-derived blood perfusion maps can identify the onset of both the arterial and venous occlusion event as is evident by the lower value of blood perfusion map throughout the imaged palm region during arterial and venous occlusion. However, there are significant inter-participant variations in the estimated blood perfusion as is evident from the varying scale of the color-map for each participant. These variations are primarily due to varying level of melanin in the epidermal layer of the skin in participants of varying skin tones. To compensate for this variability, we performed a per-participant temporal normalization of the perfusion estimate by dividing the perfusion estimate with the mean perfusion in the first 20 second of the experiment.

For the results that follows, we considered the full region-of-interest (ROI) on the palm excluding the fingers to obtain an estimate of camera-based perfusion and used the perfusion index measurements from the index finger of the same hand for contact-based perfusion estimate. Figure 2(c,d) shows the Box and Whisker plot for the normalized perfusion estimate obtained using (c) PulseCam and (d) PulseOx at different stages of the arterial and venous occlusion experiment for all the 12 participants. Clearly, both the PulseCam and the PulseOx shows large difference in normalized perfusion during the arterial occlusion experiment. However, only PulseCam showed substantial difference in normalized perfusion estimate during different stages of the venous occlusion experiment. Whereas, the perfusion index measured using the PulseOx shows much higher overlap between different stages of venous occlusion.

#### Incremental vascular occlusion study

To further explore the potential of PulseCam in detecting even smaller changes in blood perfusion associated with small change in occlusion pressure, we conduct a second study, an *incremental vascular occlusion study*, on 15 *additional* healthy participants (10 male; 5 female) of varying skin tones (Fitzpatrick scale I to VI). In this incremental vascular occlusion experiment, we sequentially apply BOP of 10 mmHg, 20 mmHg, 30 mmHg … up to 20 mmHg higher than the participant’s systolic pressure. The experimental protocol we follow for incremental vascular occlusion is summarized in Fig. 2(f).

The experimental setup for the incremental vascular occlusion study is shown in Fig. 2(e). Here, we video record both the palms, but we only perform the sequential blood flow occlusion in one of the palms — the occlusion palm (O-Palm), by wrapping a pressure cuff on the upper arm. The other palm, the non-occlusion palm, is referred to as the reference palm (R-Palm). For comparisons with contact-based perfusion index measurements (i.e. PI), we placed MightySat Fingertip PulseOx from Masimo^37^ on the middle finger of each palm (O-Palm and R-Palm). More details about the experimental setup and the protocol is provided in Supplementary S.6.

During the incremental vascular occlusion experiment, we intend to change the blood perfusion in the palm gradually. However, the blood perfusion can also simultaneously change due to changing metabolic demand of the tissue or due to the body’s reaction to external temperature, or even due to breathing. These confounding factors are outside our experimental design and can lead to unintended variations in blood perfusion during the incremental vascular occlusion experiment. To cancel out these confounding factors of blood flow variations, we propose to compute a ratio of perfusion estimate at each time instant between anatomically symmetric parts of the O-Palm and the R-Palm. We call such a ratio as *spatial perfusion ratio*. In Supplementary S.6.1, with the help of preliminary experiments, we show that the spatial perfusion ratio can cancel, to a large extent, the confounding factors of perfusion variations, and be selective to the differential factor of perfusion variations between the two palms, i.e., the induced incremental vascular occlusion in the O-Palm that we are interested to measure. We follow similar processing steps to obtain perfusion index ratio from the MightySat Fingertip PulseOx attached to the index finger of the O-palm and the R-palm. Specific steps we followed for processing the video and the PulseOx data are also summarized in Supplementary S.6.4.

As shown in Fig. 2(g), we selected a pair of ROIs on the palm (green) and another pair on the index finger (red) much closer to where we placed the MightySat pulse oximeter for further analysis. During the incremental vascular occlusion experiment, we computed the spatial perfusion ratio in the two pairs of selected ROIs from the camera and compared them with the MightySat perfusion index ratio. We find that PulseCam-derived perfusion estimate is much more sensitive in detecting perfusion changes associated with partial blood flow occlusion induced by BOP between 10–70 mmHg. Whereas the contact-based perfusion index measured using a MightySat PulseOx do not show any appreciable reduction in normalized perfusion ratio due to BOP between 10–70 mmHg and can only register a reduction when the BOP is much higher between 80–120 mmHg also shown in Fig. 2(g). We did not observe much difference between the palm and the index finger ROI, and both regions are equally sensitive to small changes in perfusion pressure.

Thus, the incremental vascular occlusion study confirms that PulseCam can reliably detect partial blood flow occlusion whereas a contact-based PulseOx may not necessarily show any appreciable reduction in the pulsatile perfusion during partial occlusion. Hence, PulseCam could potentially be used to detect partial or venous occlusion, e.g., during microvascular flap surgeries venous occlusion occurs more often than arterial occlusion, and is the primary cause of flap failures^38,39^.

### Comparing PulseCam with an infrared thermal camera

To compare PulseCam and infrared thermography, we perform incremental vascular occlusion experiment on 9 participants (5 male and 4 female) of varying skin tones (Fitzpatrick Scale I to VI) and record their palm videos using both a color camera as well as an infrared thermal imaging camera simultaneously. We use a scientific-grade FLIR T1030sc thermal camera with sensitivity <20 mK operated at 10 fps to record the video of both the palm during the occlusion experiment. Therefore, we simultaneously obtain spatial blood perfusion maps using PulseCam as well as spatial temperature map using the infrared thermal camera from both the O-palm and the R-palm during incremental vascular occlusion.

**Figure 3:**
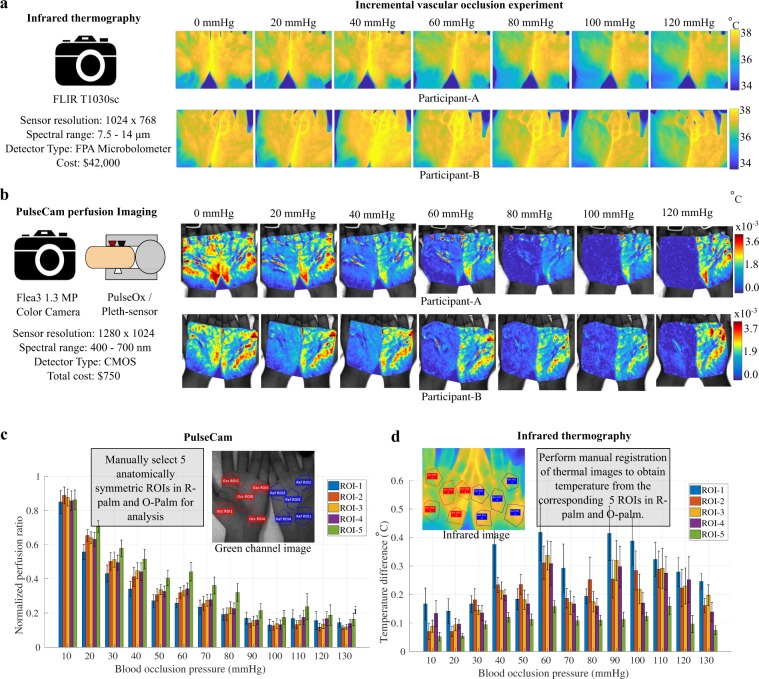
Comparing performance of PulseCam and infrared thermography: (**a**) Spatial map of skin temperature for two participants (A and B) obtained using infrared thermal camera, and (**b**) spatial map of pulsatile blood perfusion obtained using PulseCam for the same two participants (out of 9 in total) at different stages of incremental vascular occlusion experiment; PulseCam provides higher spatial resolution blood perfusion map compared to an infrared thermal camera, (**c**) (left) normalized perfusion ratio at different levels of vascular occlusion obtained using PulseCam; (**d**) (right) corresponding temperature change obtained using the infrared thermal camera for each of the five ROIs at different occlusion pressure levels aggregated for all the 9 participants. Clearly, PulseCam shows a gradual reduction in the pulsatile perfusion as the occlusion pressure is increased whereas temperature difference shows an irregular variation across all the 5 regions of the palm.

  Figure 3(a) shows the spatial map of temperature obtained using the infrared thermal camera for two participants (A and B), and Fig. 3(b) shows the corresponding spatial map of blood perfusion obtained using PulseCam for the same 2 participants (out of 9) during different stages of the incremental vascular occlusion. Clearly, PulseCam provides higher spatial resolution blood perfusion maps compared to a thermal camera as the heat transfer to the skin surface due to blood flow underneath the skin is diffused in nature. Also, different levels of vascular occlusion are much more pronounced in blood perfusion maps obtained using PulseCam compared to temperature maps obtained using the infrared thermal camera.

For further analysis, we manually select 5 anatomically symmetric ROIs in the O-Palm and R-Palm image obtained from the color camera and the infrared camera, i.e., we perform manual registration between the two cameras. To reject the confounding factors of blood perfusion variations, we compute spatial perfusion ratio between selected ROIs in the O-palm and the R-palm as discussed earlier. Similarly, we compute the temperature difference between the corresponding ROIs in the O-palm and the R-palm to cancel out confounding factors of temperature variations that are not linked with the incremental vascular occlusion experiment. We then compute the maximum temperature change between consecutive release phase and the occlusion phase to estimate the maximum change in temperature associated with the level of occlusion pressure. Figure 3(c) (left) shows that the normalized perfusion ratio obtained using PulseCam follows a gradual reduction in all the 5 ROIs in the palm with increasing occlusion pressure for 9 study participants. On the other hand, Fig. 3(d) (right) shows that the temperature change varies irregularly with increasing occlusion pressure for each of the selected 5 ROIs in the palms of the 9 participants in the study.

To evaluate the occlusion detection performance of the two modalities, we train a decision tree regression model to identify the level of occlusion given the perfusion measurements from the PulseCam or temperature measurements from the thermal camera. We split all the participants randomly into a training set (6) and a test set (3) and use either the normalized perfusion ratio in the 5 ROIs or the temperature change in the 5 ROIs at different occlusion pressure as the feature set to learn the decision tree regression model. The mean absolute deviation (MAD) in detecting occlusion pressure level when using PulseCam-based decision tree model is 16.0 mmHg (test performance), whereas the MAD for the thermal camera based model is 33.8 mmHg (test performance). These comparisons highlight that PulseCam is better in detecting micro-circulatory blood perfusion changes associated with incremental vascular occlusion compared to a much more expensive infrared thermal camera. Further, there is a latency of 24–30 seconds in measuring temperature change associated with occlusion using a thermal camera whereas the corresponding latency in measuring normalized perfusion change using PulseCam is only between 7–12 seconds based on the window size (2*T* + 1) chosen for analysis. So, PulseCam has 2–3 times better response time in detecting blood perfusion occlusion compared to an infrared thermal camera.

### Perfusion monitoring during surgery

To demonstrate the utility of PulseCam in measuring perfusion changes in a clinical setting, we conduct a pilot observational study on 10 patients undergoing surgery in which general anesthesia is used. In this experiment, we want to evaluate how reliably can PulseCam detect blood perfusion changes associated with the vasodilation from only the palm video recording of patients in an operationally challenging surgery room setting. For this study, we approach the patients before the surgery to request their participation. We obtained informed consent from all the participants. The study was approved by the institute review board for Baylor College of Medicine (Protocol number: H-42182).

**Figure 4:**
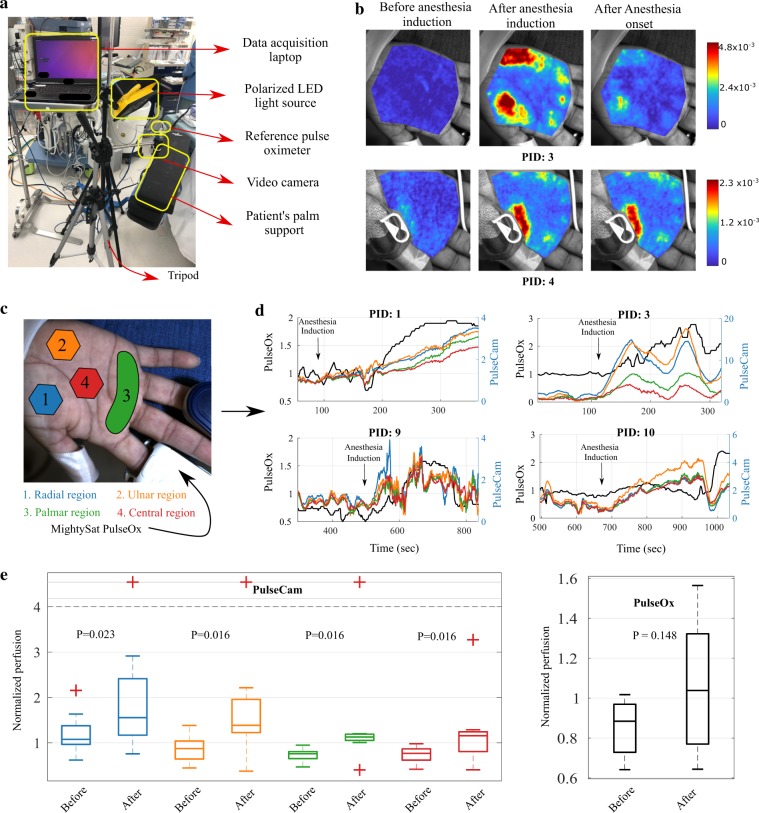
Perfusion monitoring during surgery using PulseCam: (**a**) PulseCam experimental setup used in the surgery room to monitor perfusion in the palm region before and after anesthesia induction, (**b**) spatial blood perfusion map in the palm region at different stages of anesthesia induction during the surgery, (**c**) Four regions (ROIs) in the palm selected based on the arteries that supply blood (to the regions); contact-based PulseOx is attached to the index finger, (**d**) temporal trend of normalized perfusion in the four ROIs of the palm obtained using PulseCam (colored) or using the contact-based PulseOx (black) for four (out of ten) patients during the surgery, (**e**) Box plot showing the perfusion estimate obtained using PulseCam (left) from different ROIs of the palm and using Masimo’s MightySat PulseOx (right); PulseCam can detect a statistically significant increase in perfusion before and after anesthesia induction (*p* < 0.05) from all the four ROIs in the palm, whereas a PulseOx does not register a significant increase in perfusion due to anesthesia induction (*p* = 0.148); we use Wilcoxon signed ranked test to evaluate the statistical significance of change.

  Figure 4(a) shows the experimental setup used in the surgery room which consists of a video camera, a reference PulseOx, a Masimo MightySat PulseOx (for comparison) and a data acquisition laptop. We also place polarizers both on the camera as well as on the light source to minimize specular reflection. However, we could not restrict unpolarized ambient light to fall on the palm as we did not have any control on the ambient operating room light settings as are required for the clinical care of the patients by the surgery team. We recorded the patient’s palm videos from the start of the surgery before anesthesia induction up to the time of the onset of the anesthesia as determined by the attending anesthesiologist. We also simultaneously record the perfusion index measurements using a contact-based Masimo MightySat^37^ PulseOx for comparison. More details about the experimental setup and the data processing steps we followed are summarized in Supplementary S.7.

Figure 4(b)  shows the spatial blood perfusion map in the palm region for 2 (out of 10) patients at three time points: (i) just before anesthesia induction (left), (ii) 90 secs after anesthesia induction (center), and (iii) at the time of the onset of anesthesia (right). These spatial maps of blood perfusion clearly highlight that PulseCam can register an increase in peripheral perfusion due to vasodilation just after anesthesia induction. The increase in peripheral perfusion is specifically higher in the radial and the ulnar regions of the palm as can be seen from these spatial maps. The spatiotemporal analysis as well as the results obtained from all the 10 patients are detailed further in Supplementary S.7.5.

For further analysis, we consider four distinct regions in the palm based on the arteries that supply blood to those region: (i) radial region, (ii) ulnar region, (iii) palmar arch region, and (iv) central regions as highlighted in Fig. 4(c) (regions 1 to 4). To compensate for the inter-subject variability in peripheral perfusion estimate, we perform temporal normalization with the baseline perfusion estimate in the palm region during the first 20 sec of the data recording for each participant before anesthesia induction. We followed similar normalization steps for the perfusion index measured using the Masimo’s MightySat PulseOx. Figure 4(d) shows the temporal variations in normalized peripheral perfusion in the four spatial regions of the palm as well as the perfusion index (PI) measurement obtained from the Masimo MightySat PulseOx attached to the patient’s finger (shown in black) for 4 (out of 10) patients.

To compare the normalized perfusion before and after anesthesia induction across patients, we choose a 60 seconds time window just before the anesthesia induction and a 60 seconds time window after 30 seconds of anesthesia induction. To evaluate the statistical significance of changes in the normalized perfusion estimate, we use the Wilcoxon signed rank test as we can not assume that the measurements are normally distributed and consider *p* < 0.05 as the condition for statistical significance for this pilot observational study. During this analysis, we have to reject data from two patients as for one of the patients there was no anesthesia induction during the surgery (clinical condition of the patient changed precluding administration of general anesthesia), and for the other patient, the reference pulse oximeter lost contact with the index finger resulting in corrupted data. Figure 4(e) shows the Box and Whisker plot for the mean perfusion estimate obtained from different regions of the palm using PulseCam (left) and the MightySat PulseOx (right) for the remaining 8 patients having valid data recordings. The perfusion estimated using PulseCam from all the different regions of the palm shows a statistically significant increase in perfusion after anesthesia induction with *p* < 0.05. However, the difference in perfusion index measured at the index finger using the Masimo MightySat PulseOx was not statistically significant with *p* = 0.148.

This pilot observational study highlights both the opportunities as well as the challenges associated with using PulseCam in a clinical setting. On the one hand, we encounter operational challenges such as loss of contact of the reference PulseOx  challenges associated with placement of the prototype system in an already crowded surgery room setting, unpolarized and flickering ceiling light that we could not control, etc. On the other hand, we also showed that our PulseCam approach is robust to several of these operational challenges, and we could establish that the camera-based perfusion estimate obtained using our algorithm has a better chance of detecting blood perfusion increase associated with anesthesia induction (due to vasodilation) compared to a contact-based PulseOx.

## Discussion

In this paper, we introduced PulseCam as a new blood perfusion imaging modality that combines the video recording of skin surface or internal tissue with a reference blood volume waveform to reliably obtain spatiotemporal pulsatile blood perfusion map. We limited the evaluation of PulseCam and its comparisons with competing methods to only the palm region because of the ease of access and experiment simplicity. However, PulseCam methodology can easily be extended to obtain blood perfusion maps from other body parts such as the face, foot, and the abdomen. Additionally, PulseCam can also potentially be used to obtain blood perfusion maps from internal tissues using existing camera attached to an endoscope or a laparoscope with minimal modification. Hence, PulseCam could be employed in minimally invasive surgeries to visualize blood perfusion, e.g., for identifying anastomotic failures after surgical interventions such as bowel resection^40^ and intestinal surgeries^41^, and also for localizing cancerous tissue that usually shows distinctly different blood perfusion signatures^42^.

**Figure 5:**
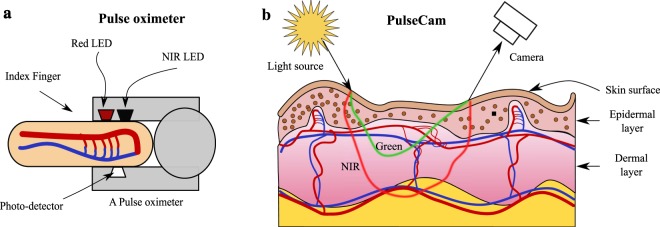
Differences in interaction of light with tissue in PulseOx and PulseCam: (**a**) Conventional PulseOx utilizes a transmission mode geometry such that the measured light interacts with both the surface micro-vessels as well as more prominent arteries and veins that resides much deeper, (**b**) PulseCam utilizes a reflectance mode geometry where light only interacts with surface micro-vessels before it gets back-scattered.

Based on both the blood flow occlusion experiments as well as the perfusion monitoring during surgery experiment, we have demonstrated that PulseCam has higher sensitivity in detecting micro-vascular blood perfusion changes compared to a PulseOx derived perfusion index. We believe that this difference in sensitivity of camera-based and contact-based modalities is due to the difference in the effective penetration depth of the recorded light into the skin. A contact-based PulseOx is usually based on a transmission geometry (e.g., Masimo’s MightySat used in our study) where the light source and the photo-detector are on opposite sides of the tissue or organ, e.g., the finger. Therefore, the measured light is primarily modulated by blood volume changes in thicker arteries that reside much deeper (See Fig. 5(a)). On the other hand, PulseCam is based on reflection geometry where the light source and the camera are on the same side of the skin surface. Therefore, the recorded light intensity is only modulated by the blood volume change in the smaller micro-vessels closer to the skin surface (See Fig. 5(b)). Even a small blood occlusion pressure, e.g., between 10–40 mmHg, can result in significant reduction in the pulsatile blood perfusion in these tiny vessels at the surface, and this reduction can be easily picked up by the camera, but not by a PulseOx, thus possibly explaining the higher sensitivity of PulseCam in detecting partial blood flow occlusion. However, being sensitive to only the microvascular blood perfusion changes is also a limitation of PulseCam as it cannot be used to image the flow of blood in deeper layers of the tissue. A near-infrared (NIR) camera, instead of a color camera, can be used to possibly extend the depth of blood perfusion measurements as the near-infrared light penetrates slightly deeper into the tissue compared to visible light owing to its lower absorption coefficient (See Fig. 5(b)). Thus, one can potentially combine PulseCam’s perfusion estimates from several color channels, e.g., red, green and near-infrared, to obtain a tomographic view of pulsatile blood perfusion map at different depth underneath the skin or internal tissue, and has been shown to be feasible recently based on experimental studies on tissue^43^.

Apart from proposing robust signal recovery and optical flow techniques for blood perfusion imaging, we have also studied the impact of camera measurement noise on estimating pulsatile pulsatile blood perfusion maps (see Supplementary S.4 for more details). In conventional (biomedical) imaging and computer vision applications, the relative magnitude of noise *σ*_*w*_ compared to the signal of interest *b* (usually the mean pixel intensity) is small, i.e., *σ*_*w*_/*b* ≈ 0.01. Whereas for blood perfusion imaging the relative magnitude of measurement noise compared to the pulsatile amplitude of subsurface reflection *a* is much higher (*σ*_*w*_/*a* ≈ 10). So, there is a need to quantify the impact of camera measurement noise onto perfusion estimate. For this, we developed an operational equation (Equation S4) that explicitly shows how standard deviation of perfusion estimate is related to camera’s measurement noise. This equation also highlights how one can trade-off spatial and temporal resolution of obtained blood perfusion maps to reduce the standard deviation of the perfusion estimate (See Fig. S6 for more details). Based on this analysis, we chose the spatial and temporal averaging parameters and lighting parameters to keep the expected percent perfusion error due to camera measurement noise to be below 10% for all of the participants in our study.

One of the critical algorithmic innovation in PulseCam is the multi-sensor robust blood perfusion estimator to reliably estimate blood perfusion maps in the presence of motion artifact and outliers in the video recording of the skin surface. However, this multi-sensory approach assumes reliable access to a reference blood volume waveform recorded using a contact-based wearable optical sensor. Thus, PulseCam can potentially suffer downtime and produce erroneous results if the reference blood volume waveform is corrupted due to motion artifacts or loss of contact with the skin surface. To guard against such corruption, we devised a reference pulse validity test that automatically detects sensor saturation and motion artifacts in the contact-based PulseOx, and flags the corresponding reference samples as erroneous. If a large number of contiguous samples are corrupted, then we do not compute the perfusion estimate over the corresponding time window and consider it as algorithmic downtime. More details about the reference validity test is provided in Supplementary S.7.4. For our in-lab comparisons across different modalities, the downtime was 0%, whereas, for the surgery room data collection, the overall downtime was 12% as we also had to entirely reject one patient data (PID: 3) in the surgery room dataset because of the corrupted reference pulse waveform; this downtime can easily be reduced as our setup is experimental and no clinical staff was assigned to monitor PulseCam operation or hardware operation, beyond merely turning it on.

Blood perfusion at any specific skin site depends on multiple physiological factors such as patient’s circulatory state, vasodilation and vasoconstriction, and body`s reaction to external temperature (neurohumoral control), etc. Also, there could be multiple pathophysiological factors, e.g., arterial or venous occlusion, peripheral arterial disease, circulatory shock, or melanoma that can cause changes in peripheral perfusion. To disambiguate these diverse factors of blood perfusion variations using only one-dimensional temporal information of perfusion obtained using a contact-based sensor such as NIRS or a PulseOx or an LDF sensor is challenging, and therefore these point modalities are generally used only as a trend monitoring tool^9,44^ in clinical practice. Instead, PulseCam provides high dimensional spatial and temporal blood perfusion information, and thus potentially opens up the opportunity to combine and collate both spatial as well as temporal dependence to disambiguate diverse factors of blood perfusion variations. In this paper, we specifically demonstrated that computing ratios of peripheral perfusion estimated from two anatomically symmetric location helps to cancel out common factors of perfusion variations, and magnify the differences in perfusion at the two measurement sites. We consider PulseCam as a new imaging tool that opens up even more such possibilities of analyzing the circulatory status of patients, e.g., computing spatial perfusion index ratio between a body’s extremity, such as a finger, and a central location, such as the forehead, can provide clearer insight into the extent to which circulation to non-vital peripheral site reduced compared to vital organs during a circulatory shock in an ICU. Similarly; computing perfusion ratio between the foot and the palm may be helpful in understanding the extent to which the microcirculation in the lower extremity is compromised due to diabetes and vascular disease, e.g., critical limb ischemia (CLI), and can potentially be used as a replacement for the ankle-brachial index (ABI) as well.

## Methodology

The experimental protocols followed during the arterial and venous occlusion study and the incremental vascular occlusion study were approved by Rice University’s Institute Review Board (Protocol number 928192-1). The experimental protocol followed during the general anesthesia perfusion monitoring study was approved by the institute review board for Baylor College of Medicine (Protocol number: H-42182. Informed consent were obtained from all the participants before the study. All the methods were carried out in accordance with the relevant guidelines and regulations.

Let us denote the video recording of the skin surface by $I({\overrightarrow{x}}_{{\rm{c}}{\rm{a}}{\rm{m}}},t)$ where ${\overrightarrow{x}}_{{\rm{c}}{\rm{a}}{\rm{m}}}=({x}_{{\rm{c}}{\rm{a}}{\rm{m}}},{y}_{{\rm{c}}{\rm{a}}{\rm{m}}})$ is the camera’s pixel coordinate and *t* is the time of the measurement. Simultaneously, in PulseCam, we also record the reference pulse waveform *p*(*t*) using a wearable optical sensor such as a contact-based pulse oximeter placed anywhere on the body. Our aim is to reliably estimate the blood perfusion map ${{\hat{A}}}(\overrightarrow{x},t)$ by combining the video recording of the skin surface and the contact-based reference pulse waveform recording.

**Figure 6:**
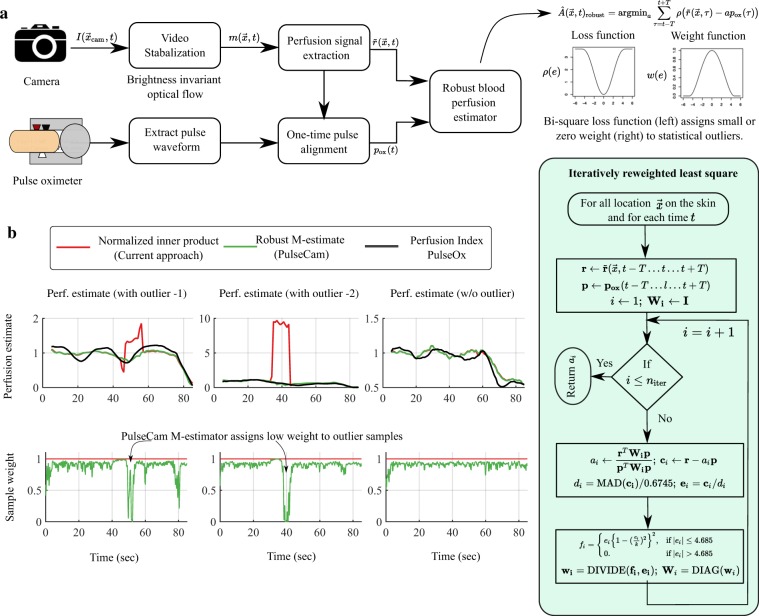
PulseCam Robust multi-sensor blood perfusion estimator: (**a**) PulseCam block diagram to combine the video recording of the skin surface and a reference pulse waveform to robustly estimate blood perfusion map; we use iterative re-weighted least square to minimize the non-linear bi-square loss function as detailed in the inlet flowchart (right), (**b**) shows two instances where perfusion estimate using the normalized inner product (red) is corrupted due to outliers, whereas the perfusion estimate using the PulseCam’s M-estimator (green) is robust to outliers, and also a third instance (right) where there are no outliers, and both the normalized inner product (red) and M-estimator (green) provide similar perfusion estimate.

We will first outline our video stabilization strategy needed to establish a correspondence between every camera pixel ${\overrightarrow{x}}_{{\rm{c}}{\rm{a}}{\rm{m}}}=({x}_{{\rm{c}}{\rm{a}}{\rm{m}}},{y}_{{\rm{c}}{\rm{a}}{\rm{m}}})$ and the physical location $\overrightarrow{x}=(x,y)$ on the skin surface at each time instant *t* in the presence of motion. Next, we will describe the processing steps needed to extract the small blood perfusion related signal from the stabilized video in presence of measurement noise and quantization error. Finally, we will elaborate the robust blood perfusion estimator that we develop to combine the blood perfusion related signal extracted from the video and the reference pulse waveform to obtain blood perfusion maps ${{\hat{A}}}(\overrightarrow{x},t)$ at each skin location $\overrightarrow{x}$ and time *t* in presence of motion artifacts and outliers in the video recording. A simplified model of the blood perfusion signal and camera’s measurement noise relevant for this algorithm development is described in Supplementary S.1. We have summarized the different processing steps involved in the PulseCam methodology in Fig. 6.

### Video stabilization in blood perfusion imaging

A correspondence between camera pixel ${\overrightarrow{x}}_{cam}=({x}_{{\rm{c}}{\rm{a}}{\rm{m}}},{y}_{{\rm{c}}{\rm{a}}{\rm{m}}})$ and the physical location on the skin surface $\overrightarrow{x}=(x,y)$ at each time instant *t* can be established with the help of an image warping function *W*^*t*^. The warping function *W*^*t*^ can be obtained by using standard optical flow algorithms^34,35^. However, to find the correspondence, most optical flow algorithm assumes brightness constancy i.e. they assume that *I*(*x*_cam_, *y*_cam_, *t*) = *I*(*x*_cam_ + *v*_*x*_, *y*_cam_ + *v*_*y*_, *t* + 1) where $\overrightarrow{v}=({v}_{x},{v}_{y})$ is the unknown optical flow vectors between frames *t* and *t* + 1 and is required to obtain the warping function *W*^*t*^. However, in blood perfusion imaging, the gray-scale pixel value at any skin site also changes due to the change in the blood volume underneath the skin surface making the brightness constancy assumption in standard optical flow algorithms inappropriate for blood perfusion imaging applications.

Though, at first sight, this might appear to be a small issue as the pixel intensity variations due to blood volume change is really small. However, with the help of a realistic blood perfusion simulator, in Supplementary S.2, we have shown that the error in the estimated blood perfusion maps due to the inapplicable brightness constancy assumption in existing video stabilization approaches^27^ can be as large as 33%, and therefore cannot be overlooked. In PulseCam, we resolve this issue by adopting an optical flow algorithm that does not require the brightness constancy assumption, and therefore, is better suitable for video stabilization in blood perfusion imaging.

#### Brightness invariant optical flow algorithm

For brightness invariant optical flow estimation, we choose zero-mean Normalized Cross Correlation (NCC) feature descriptor^45^ as it is invariant to linear brightness changes between two consecutive frames. Let us consider a small neighborhood (denoted as ${{\mathcal{N}}}_{q}$) of size (2*L* + 1) × (2*L* + 1) pixel block around a point $\overrightarrow{q}=({q}_{x},{q}_{y})$ at time *t*. The NCC distance between this patch and a nearby patch centered at $\overrightarrow{q}+\overrightarrow{w}=({q}_{x}+{w}_{x},{q}_{y}+{w}_{y})$ in the next frame at time *t* + 1 is given by

1 $$\,{\rm{N}}{\rm{C}}{\rm{C}}\,(\overrightarrow{q},\overrightarrow{w})=\frac{1}{| {{\mathcal{N}}}_{q}| }\sum _{s\in {{\mathcal{N}}}_{q}}\left\{1-\frac{(I(\overrightarrow{s}+\overrightarrow{w},t+1)-\mu (\overrightarrow{q}+\overrightarrow{w},t+1))\cdot (I(\overrightarrow{s},t)-\mu (\overrightarrow{q},t))}{\sigma (\overrightarrow{q}+\overrightarrow{w},t+1)\cdot \sigma (\overrightarrow{q},t)}\right\}.$$

In the above equation, $\mu (\overrightarrow{q},t)$ and $\sigma (\overrightarrow{q},t)$ are the mean and the standard deviation of the pixel intensity for patch ${{\mathcal{N}}}_{q}$ around the point $\overrightarrow{q}$ at time *t*, and $\mu (\overrightarrow{q}+\overrightarrow{w},t+1)$ and $\sigma (\overrightarrow{q}+\overrightarrow{w},t+1)$ are similarly defined at time *t* + 1.

The optical flow vector $\overrightarrow{v}(\overrightarrow{q})$ for the point *q* between the two frames can then be determined by minimizing the NCC distance, i.e. $\overrightarrow{v}(\overrightarrow{q})=\arg {\min }_{\overrightarrow{w}}\,{\rm{N}}{\rm{C}}{\rm{C}}\,(\overrightarrow{q},\overrightarrow{w})$. Subtracting the mean pixel intensity from each patch to compute the normalized cross-correlation distance is the key computation step that makes NCC-based feature descriptor invariant to linear brightness changes, and thus suitable for optical flow estimation in blood perfusion imaging. The normalized cross-correlation function is highly non-linear, and several optimization strategies are usually employed to obtain the optical flow based on the above cost function. Here, we adopted a low time complexity implementation of these optimization strategies as described in^31^ for a fast inverse search of patch correspondence. We set the window size parameter *L* = 8 and perform this optimization with stride length of 2 px and eventually performed a variational refinement to obtain dense optical flow. Thus, by processing the images at time *t* and *t* + 1 for all such points $\overrightarrow{q}=({q}_{x},{q}_{y})$, we obtain dense optical flow vector $\overrightarrow{v}({x}_{{\rm{c}}{\rm{a}}{\rm{m}}},{y}_{{\rm{c}}{\rm{a}}{\rm{m}}},t)$. These optical flow vectors over time can be combined to perform image warping for video stabilization i.e.

2 $$m(\overrightarrow{x},t)=I({W}^{t}({\overrightarrow{x}}_{{\rm{c}}{\rm{a}}{\rm{m}}}),t),$$

where *m* is the stabilized video recording in skin coordinates $\overrightarrow{x}$ rather than the pixel coordinates ${\overrightarrow{x}}_{{\rm{c}}{\rm{a}}{\rm{m}}}$.

### Processing steps to extract blood perfusion signal

A perfusion-related signal at each skin site $\overrightarrow{x}=(x,y)$ is extracted by first spatially averaging the stabilized video over a (2*K* + 1) × (2*K* + 1) sized pixel block, i.e.

3 $$r(x,y,t)=\frac{1}{{(2K+1)}^{2}}{\sum }_{{l}_{2}=-K}^{K}{\sum }_{{l}_{1}=-K}^{K}m(x+{l}_{1},y+{l}_{2},t).$$

Then, we extract the cardiac-related pulsatile time-varying changes in skin reflectance by passing *r*(*x*, *y*, *r*) through a band-pass filter with pass-band between the physiologically relevant frequency band of [0.5 Hz–5 Hz]. This pass-band component is also referred to as AC component. Similarly, a DC component is computed by passing *r*(*x*, *y*, *t*) through a low-pass filter with cutoff frequency of 0.3Hz. Then, we compute a normalized AC/DC ratio at each skin site (*x*, *y*) to make perfusion measurements invariant to intensity of incident light i.e.

4 $$\widetilde{r}(\overrightarrow{x},t)=\frac{{r}^{{\rm{A}}{\rm{C}}}(x,y,t)}{{r}^{{\rm{D}}{\rm{C}}}(x,y,t)}.$$

This step is essential as the illumination intensity can vary over the skin surface (curvature causes lambertian shading) as well as the illumination can change from one experiment to the next or can be changed by merely changing the location of the light source.

Based on our simplified blood perfusion signal and noise model presented in Supplementary S.1, the normalized signal $\widetilde{r}(\overrightarrow{x},t)$ equals

5 $${\widetilde{r}}_{\lambda }(\overrightarrow{x},t)={A}_{\lambda }(\overrightarrow{x},t)p(t)+n(\overrightarrow{x},t),$$

where ${A}_{\lambda }(\overrightarrow{x},t)=\frac{{a}_{\lambda }(\overrightarrow{x},t)}{{\bar{b}}_{\lambda }(\overrightarrow{x})}$ is the ratio of pulsatile perfusion amplitude *a* to the averaged surface reflectance component *b* and will be considered as a measure of blood perfusion amplitude, *p*(*t*) is the underlying pulse signal and $n(\overrightarrow{x},t)$ is the camera noise after performing the “AC over DC” step. If the spatial averaging is done over pixel block of size 2*K* + 1 such that (2*K* + 1)^2^ > 16, then the noise can be modeled as normally distributed based on the central limit theorem.

The blood perfusion amplitude can be obtained using a maximum likelihood estimator which in this case simplifies to a least square normalized inner-product between $\widetilde{r}(\overrightarrow{x},t)$ and the underlying pulse waveform *p*(*t*), i.e.

6 $${\hat{A}}{(\overrightarrow{x},t)}_{{\rm{M}}{\rm{L}}}=\frac{{\sum }_{\tau =t-T}^{t+T}\widetilde{r}(\overrightarrow{x},\tau )p(\tau )}{{\sum }_{\tau =t-T}^{t+T}{p}^{2}(\tau )}=\frac{\widetilde{{\bf{r}}}{(\overrightarrow{x},t)}^{T}{\bf{p}}(t)}{{{\bf{p}}}^{T}(t){\bf{p}}(t)},$$

where the bold-faced notation is used to denote vectors of length (2*T* + 1) samples around time *t*. This ML-estimator for blood perfusion is unbiased and consistent but assumes that we know the underlying pulse signal *p*(*t*).

In the camera-only approach^24,46^, the pulse signal *p*(*t*) is also estimated from the video recording by averaging the pixel intensities from a user selected ROI on the imaged skin surface. However, this approach usually suffer from the challenge of correlated noise and motion artifacts as both the camera-derived reference waveform *p*(*t*) and the normalized perfusion-related signal $\widetilde{r}(\overrightarrow{x},t)$ can be similarly corrupted, e.g. due to motion. Also, the camera-only approach suffers from the challenge of gross errors when the skin region under investigation is not well perfused, and therefore, extracting a reliable reference pulse waveform *p*(*t*) from only the video recording is not feasible.

### PulseCam robust blood perfusion estimator

In PulseCam methodology, we improve the reliability of blood perfusion estimate in two ways: (i) we use a contact-based pulse oximeter placed at a well-perfused reference site anywhere on the body as a source of a reliable reference pulse waveform *p*_ox_(*t*) as opposed to estimating the pulse *p*(*t*) waveform from the video recording, and (ii) instead of using a least square normalized inner product perfusion estimator, we use a robust M estimator^47^ that automatically reject outliers in the video recordings and estimate blood perfusion maps in a reliable way in presence of motion artifact and measurement noise. Here, we will first describe the processing steps needed to obtain the reference pulse waveform from the pulse oximeter and then provide details about the robust perfusion estimation.

The unprocessed output from the pulse oximeter is first band-pass filtered between the physiologically relevant frequency band of [0.5 Hz–5 Hz], and then down-sampled to match the sampling rate (or frame rate) of the video camera. Then, we estimate the envelope of the down-sampled pulse waveform using the absolute value of its Hilbert transform, and normalize the down-sampled signal by the computed envelope to obtain the reference pulse waveform *p*_ox_(*t*).

The reference pulse waveform *p*_ox_(*t*) obtained using the pulse oximeter may not be temporally aligned with the pulse waveform *p*(*t*) at the camera measurement site due to the physiological difference between the time of arrival of the blood volume pulse at the measurement site (e.g. foot) and the reference site (e.g. index finger) (a.k.a. Pulse Transit Time). Further, there could also be system-level delays between the camera and pulse oximeter recordings. To compensate for these delays, we perform a one-time calibration to estimate the overall time delay *d*_ox_ between the reference pulse oximeter waveform *p*_ox_(*t*) and pulse waveform at the camera measurement site *p*(*t*). As *p*(*t*) is unknown, we first estimate it from the video recordings of the skin surface using^48^, and then estimate the delay between the camera and the pulse oximeter using

7 $${d}_{{\rm{ox}}}=\text{arg}\,\mathop{\max }\limits_{\tau }(\widehat{{\bf{p}}}{(t)}^{T}{{\bf{p}}}_{{\rm{ox}}}(t-\tau )).$$

We then use the computed delay *d*_ox_ to align the pulse oximeter waveform *p*_ox_(*t*) with the video recordings.

The maximum likelihood blood perfusion estimator (Eq. (6)) is not robust to outliers in the video recording. A prominent source of error in the context of blood perfusion imaging is due to large and sudden temporal variations in the surface reflection due to even a slight movement or rotation of the skin surface. Since it is difficult to model such motion artifacts, so we treat them as outliers, and adopted a robust M-estimator^47^ methodology to automatically reject them. M-estimator can be interpreted as a weighted least square approach where a zero (or low) weight is assigned to outlier samples in the video recordings. To obtain a robust blood perfusion estimate, we minimize the M-estimator objective function

8 $$\hat{A}{(\overrightarrow{x},t)}_{{\rm{robust}}}=\mathop{\text{arg}\,\min }\limits_{a}\mathop{\sum }\limits_{\tau =t-T}^{t+T}\rho (\tilde{r}(\overrightarrow{x},\tau )-a{p}_{ox}(\tau )),$$

where *ρ* is a special type of non-linear loss function. Here, we choose the Tukey’s bi-square loss function as it assigns zero weight to statistical outliers^49^ in the video recording, also see Fig. 6 for details. The value of *k* is chosen as 4.685 which provides 95% statistical efficient to the M-estimator. This means that in the absence of any outliers, the M-estimator will produce same perfusion estimate as the ML-estimator (normalized inner product) with only 5% loss of statistical efficiency^49^. There is no closed form solution to the non-linear minimization problem in Eq. (8), and we use iterative reweighed least square^50^ approach to solve it. The detailed algorithmic steps are summarized in the image inlet in Fig. 6 (green box).

Figure 6(b)  (top-left) shows two data snapshots from the blood flow occlusion dataset as an illustration to highlight that the perfusion estimate using the normalized inner product (shown in red) can be corrupted due to outliers (e.g. large change in surface reflection), whereas the perfusion estimate obtained using the PulseCam’s M-estimator (shown in green) is robust to such localized motion artifacts. For comparison, we have also shown the perfusion index measurement simultaneously using a contact pulse oximeter (black) during the same time period. Figure 6(b) (bottom-left) also shows the corresponding weight that the M-estimator (green) assigns to each measurement sample from the video recording. Clearly, as expected, the proposed M-estimator could localize the outliers samples in the video recordings and assign them low or zero weight. Also, it is evident that the ML-estimator (normalized inner product) is not robust since it always assigns same weight (1.0) to each data sample. Figure 6(b) (top-right) also shows a third instance from the blood flow occlusion dataset where there are no outliers, and both the normalized inner product (red) and M-estimator (green) provides similar perfusion estimate. The weight assigned to each measurement sample by the M-estimator is close to 1.0, and hence M-estimator is similar to the ML estimator in absence of outliers.

### Performance improvement due to brightness invariant optical flow

To quantify the improvements in perfusion estimation due to the proposed use of brightness invariant optical flow algorithm, we first generated a synthetic blood perfusion and motion dataset that can simulate both the blood perfusion as well as translational and rotational motion of varying magnitude (See Supplemental S.3. for specific steps followed). We simulated a range of motion magnitude from no motion (stationary) to small motion (avg, motion magnitude of 0.2 cm/sec) to very large motion (upto avg. motion magnitude of 5 cm/sec). To calculate motion magnitude, we tracked several fudicial points on the skin surface in the simulated video and computed the distance traversed by these points over a 1 sec time window. Then, we averaged the distance traversed for all such points on the skin surface over the entire duration of simulation to obtain average avg. motion magnitude. To quantify error in perfusion, we define percent perfusion error (PPE) metric as

9 $$\,{\rm{P}}{\rm{P}}{\rm{E}}\,=\frac{\Vert (\hat{{\rm{A}}}(\overrightarrow{x})-(A\overrightarrow{x})\Vert }{\Vert A(\overrightarrow{x})\Vert },$$

where ${{\hat{A}}}$ is the estimated perfusion map and *A* is the ground truth perfusion map which is known in case of the synthetic perfusion video. To quantify error in optical flow, we use end point error (EPE) metric^51^.

**Figure 7:**
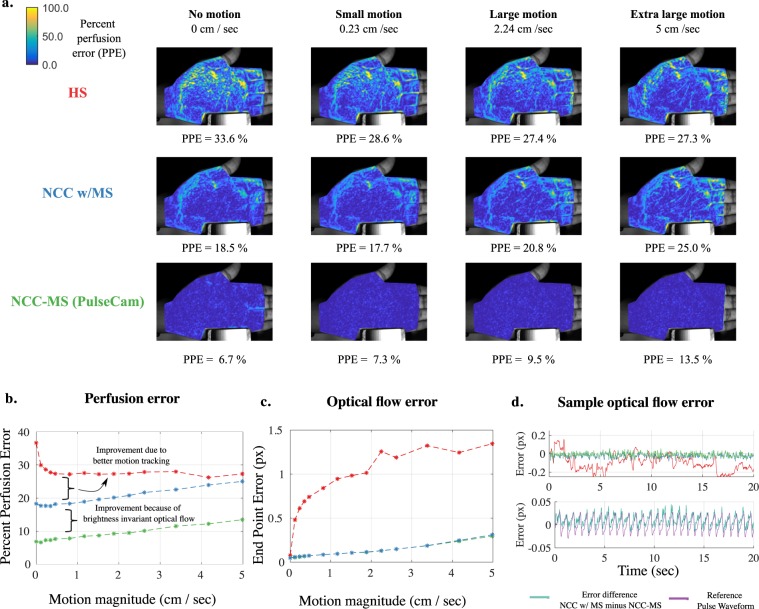
Error characteristics of three different optical flow algorithms for blood perfusion imaging: (**a**) Comparison of the spatial map of percent perfusion error (PPE) when using classical Horn Shunck (HS) optical flow, normalize cross-correlation without mean subtraction (NCC-w/MS) and NCC with mean subtraction (NCC-MS) optical flow under four simulated motion scenarios of varying magnitude, (**b**) compares the percent perfusion error (PPE) for the three optical flow algorithms evaluated in this paper when we gradually increased the motion magnitude from no motion to very large motion; our proposed approach (NCC-MS) performs 2–3 times better than other methods under widely varying motion scenarios, (**c**) compares the end-point-error (EPE) for the three optical flow algorithm; HS shows significantly (10x) higher optical flow error compared to NCC-MS or NCC w/MS, (**d**) (top) Shows sample tracking error for the three optical flow algorithm and (bottom) shows that the difference in tracking error between the NCC w/MS and NCC-MS optical flow estimate is proportional to the pulse signal (i.e. false motion) explaining why the NCC w/MS performs significantly (2 times) worse compared to NCC-MS in terms of percent perfusion error even when both algorithms have similar EPE under diverse motion scenarios.

  Figure 7(a) shows the spatial map of percent perfusion error (PPE) under motion scenarios of varying magnitude when using the classical Horn-Schunk (HS) optical flow algorithm (top-row) and our proposed Normalized Cross Correlation (NCC) approach (middle and bottom row). Here, we have compared the performance of NCC approach in two different settings — when we did not subtract the mean pixel intensity from each patch (labeled as NCC-w/MS) and when we subtracted the mean intensity from each patch to ensure invariance to linear brightness changes (labeled as NCC-MS), i.e. our proposed method Fig. 7.

During no motion scenario, the classical HS approach to optical flow shows significant error in recovering perfusion maps with PPE as high as 33.6%. The NCC w/MS shows some improvement with PPE of around 18.5%, whereas the real gain is achieved when we use NCC-MS having PPE of only 6.9%. As a baseline, even if we know the true motion trajectory of each pixel, the PPE is 4.8% owing to camera’s measurement noise which is also simulated. As further discussed in Supplemental S2, HS and NCC w/MS approach shows significantly worse performance because these optical flow algorithms imposes brightness constancy assumption even when there are small changes in the skin color due to blood volume change underneath. On the other hand, our proposed NCC-MS inherently provides invariance to linear brightness changes and perform significantly better in recovering spatial blood perfusion map.

Figure 7(b)  shows that the PPE for both NCC-MS (green) and NCC w/MS (blue) start deteriorating gradually as the motion magnitude is increased in simulation. Clearly, NCC- w/MS consistently performs at least two times worse than NCC-MS highlighting the importance of subtracting the mean pixel intensity before computing the optical flow. Interestingly, for at least simulated translational and rotational motion, PPE for HS (red) initially shows slight improvements compared to no motion scenario and then remain at a rather high level of around 28% as the motion magnitude is increased, and is therefore around 2–3 times worse than the proposed NCC-MS. Figure 7(c) shows the end-point-error (in pixels) for the three different optical flow algorithm during varying level of simulated motion. Clearly, EPE for HS is significantly (10x) worse than either NCC-MS or NCC w/MS. Therefore, the worse performance of HS under motion scenarios may be attributed to both tracking error as well as the brightness constancy assumption in HS. On the other hand, the worse performance of NCC w/MS may be solely attributed to the brightness constancy assumption as both NCC-MS and NCC w/MS show similar end point error under varying simulated motion magnitude. As an example, Fig. 7(d) (top) shows sample tracking error for a sample point on the palm during small motion simulation for the three optical flow algorithm, and (bottom) plot shows the difference in tracking error between NCC w/MS and NCC-MS. The difference in error is proportional to the underlying pulse waveform (i.e. false motion) further underscoring the role of brightness constancy assumption in the significantly worse performance of NCC w/MS compared to proposed NCC-MS.

It is important to understand that the false motion we highlighted in this work is not due to the ballistocardiogram (BCG) motion that has been studied by other researchers^26^. BCG is actually a *true-motion* and the origin of BCG is attributed to small movement of arterial walls due to the pulsatile flow of blood and can be seen around surface arteries, e.g. at the wrist. A common approach^52^ to estimate BCG motion is to compute optical flow of small patches on the skin surface in regions around the arterial walls. Based on our findings related to conventional optical flow algorithms, it will be important to revisit some of the earlier work to verify that the BCG motion other researchers observed is actually due to the motion of the skin surface i.e., is a *true-motion*, and is not due to the flawed brightness constancy assumption inherent in the optical flow algorithm used during video analysis, i.e., *false-motion* highlighted here.

## Supplementary information

Supplementary Information.

Supplementary Video 1.

Supplementary  Video 2.

Supplementary  Video 3.
