# Supplementary material for: PulseCam: a camera-based, motion-robust and highly sensitive blood perfusion imaging modality
Source: Sci Rep. 2020 Mar 16;10:4825. doi: 10.1038/s41598-020-61576-0 (PMC7075982; doi:10.1038/s41598-020-61576-0)
Supplement: Supplementary file 1 — Supplementary Information. [file 41598_2020_61576_MOESM1_ESM.pdf]

Supplemental material

PulseCam: a camera-based, motion-robust, and highly sensitive blood perfusion imaging modality

Mayank Kumar[1], James W. Suliburk[2],
Ashok Veeraraghavan[1], and Ashutosh Sabharwal[1]

[1] Electrical and Computer Engineering, Rice University, 6100 Main St, Houston, TX 77005, USA

[2] Division of General Surgery, Baylor College of Medicine, 6620 Main St, Houston, TX 77030, USA

Contents

S.1 Blood perfusion imaging signal and noise model **1**

S.2 Blood perfusion simulator **3**

S.3 Blood perfusion and motion simulation **5**

S.4 Impact of measurement noise on perfusion estimate **6**

S.5 Arterial and venous occlusion study **8**

S.6 Incremental vascular occlusion study **10**

S.7 Perfusion monitoring during surgery **13**

S.8 Supplemental videos **17**

Figures S1 to S10
Tables S1 to S2
References *(1-13)*

S.1 Blood perfusion imaging signal and noise model

Here, we present a simplified signal and noise model for blood perfusion imaging. We assume that the skin surface is illuminated with a diffused and uniform light source. Recently, [1] showed that using such a light source (homogeneous and orthogonal) significantly reduces ballistocardiography (BCG) motion artifact. Further, we assume a perpendicular polarizer configuration between the light source and the camera that helps minimize specular highlights in the video recording and further reduce BCG motion artifact [2]. Therefore, we do not consider BCG artifact as part of our signal and noise model here.

Let $m(\vec{x}, t)$ denote the light reflected from the skin location $\vec{x} = (x, y)$ at time t and is recorded by a camera. The camera-recorded light constitutes both the surface as well as the

subsurface reflection from the skin and can be modeled as

$$m(\vec{x},t) = L_\lambda(\vec{x})\Big[b_\lambda(\vec{x},t) + c_\lambda(\vec{x},t)\Big] + w(\vec{x},t), \tag{S1}$$

where $L_\lambda(\vec{x})$ is the intensity of incident light at skin location $\vec{x}$ and the wavelength λ denotes the camera's color channel. For a conventional color camera, $\lambda =$ R,G,B or for a near-infrared camera, $\lambda =$ NIR, or can be any specific wavelength range based on the optical filters used during video recording. Here, we develop a generic signal and noise model formulation which is wavelength agnostic. Similarly, our proposed PulseCam methodology can be applied to each camera channel separately. The surface reflection component is $b_\lambda(\vec{x},t)$ and the subsurface reflection component is $c_\lambda(\vec{x},t)$ and the camera's measurement noise is $w(\vec{x},t)$.

The surface reflection component $b_\lambda(\vec{x},t)$ constitutes both specular (mirror-like) reflection as well as diffused reflection. If we assume that perpendicular polarizers are used during data recording, then the specular highlights can be assumed to be small. If we assume that the skin surface does not rotate relative to the camera during the video recording then there will be minimal change in the angle between the surface normal and the direction of incident light, and consequently, the surface reflection component $b_\lambda(\vec{x},t)$ can be assumed to be constant over time, i.e. $b_\lambda(\vec{x},t) = b_\lambda(\vec{x})$. However, even a small rotation of the skin surface may cause large variations in surface reflection, and these large variations will be treated as outlier to our proposed signal and noise model as it is difficult to model such variations.

The subsurface reflection component $c_\lambda(\vec{x},t)$ is time varying due to pulsatile variations in blood volume in the arterioles embedded in the dermis and can show spatial, temporal and spectral variations. The spectral dependence is due to wavelength dependent variations in light absorption by hemoglobin and oxy-hemoglobin in the blood as well as due to variations in the penetration depth of light of different wavelengths. The subsurface reflectance component $c_\lambda(\vec{x},t)$ can be modeled as

$$c_\lambda(\vec{x},t) \approx a_\lambda(\vec{x},t)p(t - d(x)), \tag{S2}$$

where $a_\lambda(\vec{x},t)$ is the amplitude of the pulsatile variations in the subsurface reflectance, i.e. a measure of pulsatile perfusion, and $p(t)$ is the underlying pulse or blood volume waveform, and $d(x)$ is the time delay of the pulse waveform as it travels through the arterial network. Over a small region of interest (e.g. palm or foot), the delay can be considered to be constant ($d(x) = d$). For notional simplicity, we will drop the delay term. Our proposed model for subsurface reflection implicitly assumed that the shape of the pulse waveform $p(t)$ does not vary spatially, and is same for different wavelengths of light. Recently, [3] showed that such spatial and spectral dependence of the pulse waveform shape are important to consider for computing blood perfusion phase (or delay) maps, but can be neglected for estimating blood perfusion amplitude maps which is the focus of our current work.

The camera measurement noise $w(\vec{x},t)$ is comprised of photon shot noise, readout noise and quantization error. Under sufficient illumination, photon shot noise is dominant and has a Gaussian distribution and its variance is proportional to the mean incident photon flux. The readout noise is a combination of all the noise generated by camera system components and is generally modeled by a Gaussian distribution with a standard deviation dependent on the specific camera system. The quantization error is determined by the effective number of bits (ENOB) of the camera ADC. The quantization error can be modeled as uniformly distributed, and is independent of the photon shot noise and the readout noise.

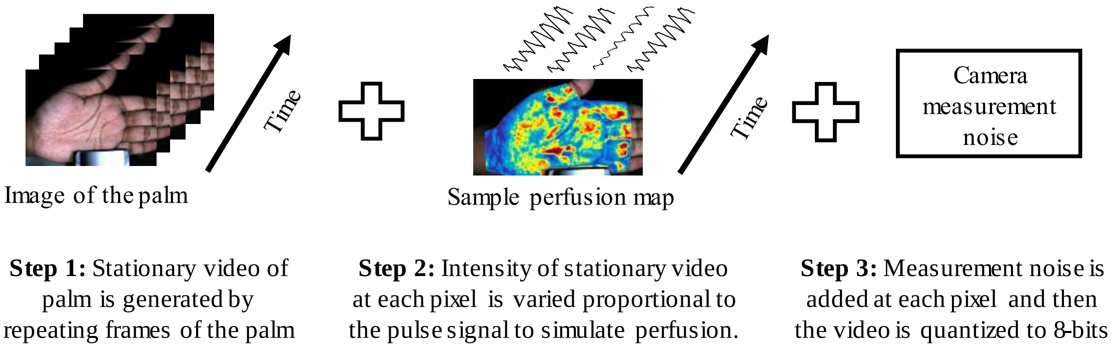

Figure S1: **Steps involved to simulate a blood perfusion video**

S.2 Blood perfusion simulator

We developed a blood perfusion simulator to better understand the impact of brightness constancy assumption during optical flow computation on the eventual perfusion estimate and evaluate the performance of existing optical flow algorithm when applied to perfusion imaging.

Figure S1 shows the steps we followed to create a blood perfusion video of the palm by directly simulating the blood perfusion signal and noise model elaborated in Equation (S1). As a first step, we repeat an image of the palm to create a static video of the palm. By construction, this video does not exhibit any motion and only have surface reflection component $b_\lambda(\vec{x})$. As a second step, we add small intensity variations at each pixel of the skin surface to simulate blood perfusion. To make our simulation realistic, we set the amplitude of the pulsatile intensity variation $a_\lambda(\vec{x})$ at each skin site equal to the value of pulsatile perfusion we measured from another sample video recording of the palm of the same participant. During this sample video recording, the participant is asked to keep the palm relatively stationary, and we recover the sample perfusion map without using any video stabilization. This sample perfusion map will be treated as a ground truth perfusion map in this simulation. As a final step, we add white Gaussian noise with standard deviation proportional to the square root of the mean pixel intensity and quantize the resultant output to 8 bits to simulate the camera measurement noise $w(\vec{x}, t)$.

We process the simulated blood perfusion video using the RealTITracker toolbox [4] to obtain optical flow vectors. The RealTITracker toolbox implements an improved version of the classical Horn-Schunck optical flow algorithm and has been used earlier to perform image registration (warping) for blood perfusion imaging applications [5]. Figure S2 shows the x and y coordinates of the estimated optical flow vector of two sample points **E** and **F** selected randomly on the skin surface (marked in yellow) as well as the ground truth optical flow vector for points **E** and **F** (i.e., no motion) as green horizontal lines. It's interesting to observe that the error in the estimated optical flow vector is proportional to the reference pulse signal $p(t)$ which is used to simulate the perfusion video and is also shown at the top right for comparison.

The magnitude of the error in the optical flow path is small and is only within ± 0.2 px at any point on the skin surface, and therefore might appear to be negligible. However, if we perform image warping using these erroneous optical flow estimate and estimate blood perfusion map using the locked-in amplification method discussed in [5], then we find that the resulting blood perfusion map is significantly corrupted. Figure S3(b) shows the estimated blood perfusion maps when using Horn-Schunck optical flow for warping, whereas Figure S3(a) shows the ground truth blood perfusion map for comparison. In Figure S3(c), we have also shown the estimated blood perfusion maps when we use the known optical flow vector (i.e., no motion)

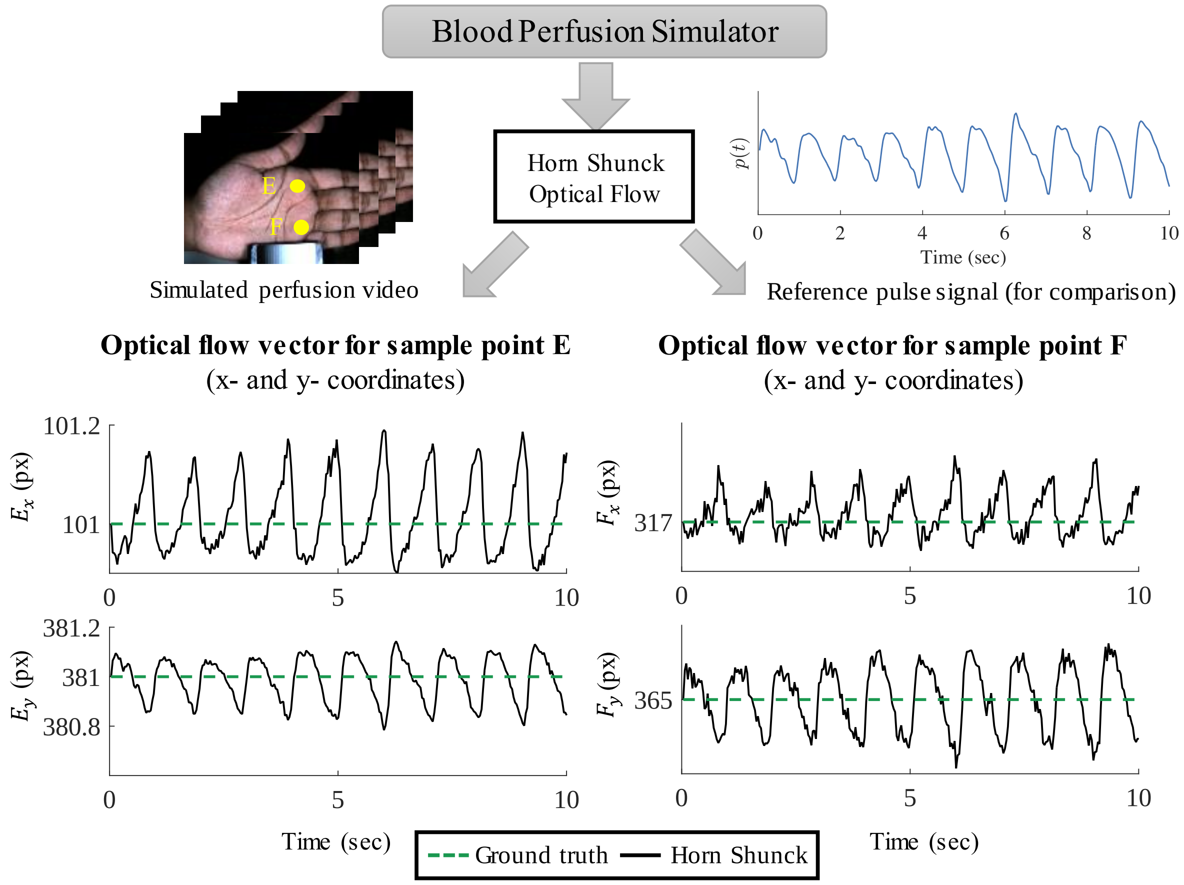

Figure S2: **Illustration of false motion with a simulated perfusion video**: Shows the x and y coordinates of the Horn-Shunck optical flow estimate of two sample points **E** and **F** (marked in yellow) on the skin surface. Also, shown are the ground truth optical flow as green horizontal line. It is interesting to note that the error in the Horn Shunck optical flow estimate is proportional to the reference pulse signal $p(t)$ which is used to simulate the perfusion video and is also shown at the top right for comparison.

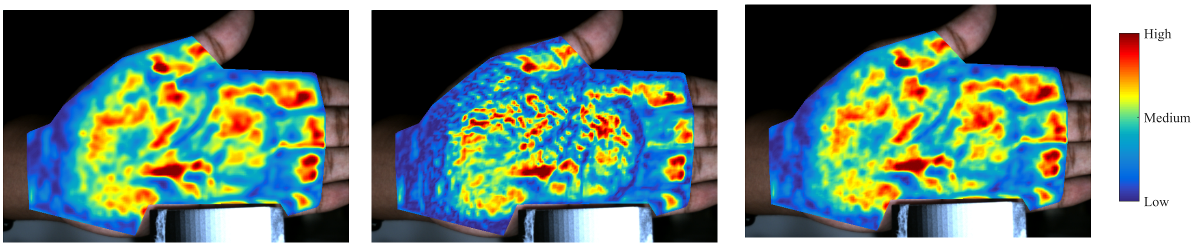

(a) Ground truth perfusion map (Known for simulated video) **Percent Perfusion Error = 0%**
(b) Estimated perfusion map when using Horn-Shunck optical flow Algo **(Percent Perfusion Error = 33.6%)**
(c) Estimated perfusion map when Using ground truth optical flow **(Percent Perfusion Error = 4.7%**

Figure S3: **Significantly corrupted blood perfusion map due to false motion**: The obtained blood perfusion map is significantly corrupted if we use Horn-Schunck optical flow algorithm for video stabilization in blood perfusion imaging. This corruption is because of the inapplicable brightness constancy assumption inherent in classical optical flow algorithm when applied to blood perfusion imaging.

for image warping. To quantify the error in the estimated perfusion maps, we compute a *percent perfusion error* (PPE) metric which we define as

$$\text{PPE} = \frac{\|(\hat{A}(\vec{x}) - (A\vec{x})\|}{\|A(\vec{x})\|}, \tag{S3}$$

where $\hat{A}$ is the estimated perfusion map and A is the ground truth perfusion map which is known in case of the simulated perfusion video. The error in the estimated blood perfusion maps if we use the Horn-Schunck optical flow estimate is 33.6% which is significantly large. Whereas the error in blood perfusion estimate if we use the known optical flow (i.e., no motion) is only 4.7%. This residual error is due to the added photon shot noise and quantization error in the perfusion simulator and is significantly smaller compared to the error due to the corrupted optical flow estimate obtained using the Horn-Schunck algorithm.

One might wonder that even in the presence of a small error on the order of $1/10^{\text{th}}$ of a pixel in the optical flow estimate, why is the error in blood perfusion maps so large? As seen in Figure S2, the optical flow vector of the sample point **E** and **F** shows small perturbations proportional to the pulse signal. These perturbations highlight the fact that Horn-Schunck optical flow algorithm is attempting to ensure brightness constancy even when there are small changes in the skin color (due to blood volume change) by perturbing optical flow vector in the x and y direction. Now, if we use these perturbed optical flow vector to perform imaging warping, then the perfusion-related signal which is essentially color-change signal at each skin site will be significantly diminished as we have already imposed a brightness constancy constraint during optical flow computation. Therefore, the error in the estimated blood perfusion maps is significantly large.

S.3 Blood perfusion and motion simulation

To simulate both motion and perfusion, we recorded two sets of videos of the palm from the same participant. The first video was recorded with limited to no motion for a duration of 2 minutes. This video was used to generate a synthetic blood perfusion simulation video of the palm having no motion as described in Appendix S.2. In the second video recording, the

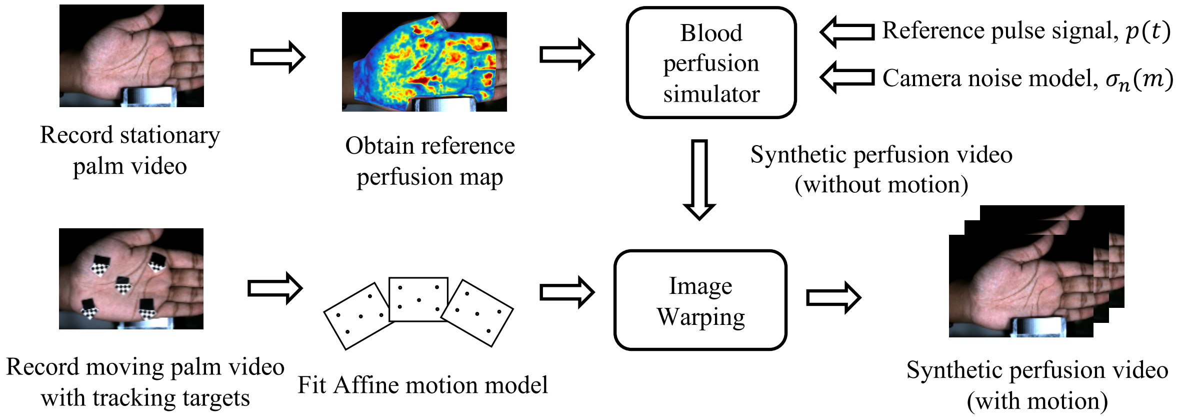

Figure S4: **Steps to generate synthetic perfusion video with motion**: First video of the palm is recorded with limited to no motion, and is used to generate a synthetic perfusion video without motion; Second video of the palm is recorded with small translational and rotational motion. We pasted 5 tracking targets on the participant's palm to reliably recover the motion without getting impacted by perfusion-related changes in the skin color. We fit an affine motion model, and used image warping to generate synthetic perfusion video with motion.

participant was asked to slowly move and rotate the palm while always remaining within the camera's field of view. While recording the second video, we also pasted 5 tracking targets on the participant's palm. The tracking target is pasted to reliably recover the motion of the palm without getting impacted by perfusion-related changes in the skin color. We used standard KLT tracking [6] to obtain the trajectory of the tracking targets as the participant move his hand. Then, we fit an affine-motion model on the recovered trajectory of the 5 tracking targets to get a realistic estimate of the motion of the palm. Finally, we used the affine motion model to warp the corresponding frames of the simulated stationary blood perfusion video to obtain a synthetic video of the palm having both blood perfusion related variations as well as rotational and translation motion. Figure S4 shows the overall steps to generate the synthetic perfusion video with motion.

S.4 Impact of measurement noise on perfusion estimate

The camera's measurement noise is dominated by the photon shot noise, readout noise, and quantization error. To measure the standard deviation of the measurement noise at different mean pixel intensity level, we recorded 300 frames of a standard Grey-scale chart using our camera setup. Any variations in the camera recording over time at any Grey-scale value (of the chart) will only be due to measurement noise. Therefore, we can directly compute the standard deviation of the measurement noise at different mean pixel intensity levels $\bar{m}$.

Figure S5 shows the variation of the standard deviation of measurement noise (σ_w) as a function of the square root of the mean pixel intensity $\sqrt{\bar{m}}$ for our camera setup. Here, all values are shown in the scale of a standard 8 bit camera ADC ($0-255$). As expected, the measurement noise increases with increasing mean pixel intensity, and can be modeled as $\sigma_n = k_1\sqrt{\bar{m}} + k_0$ where $\bar{m}$ is the mean pixel intensity. For our camera's specific settings (Exposure = 8 msec, analog gain = 0 dB), we obtained $K_0 = 0.146$ and $K_1 = 0.178$. Thus, we can use this measurement noise model to obtain an estimate of the measurement noise at each pixel during perfusion imaging.

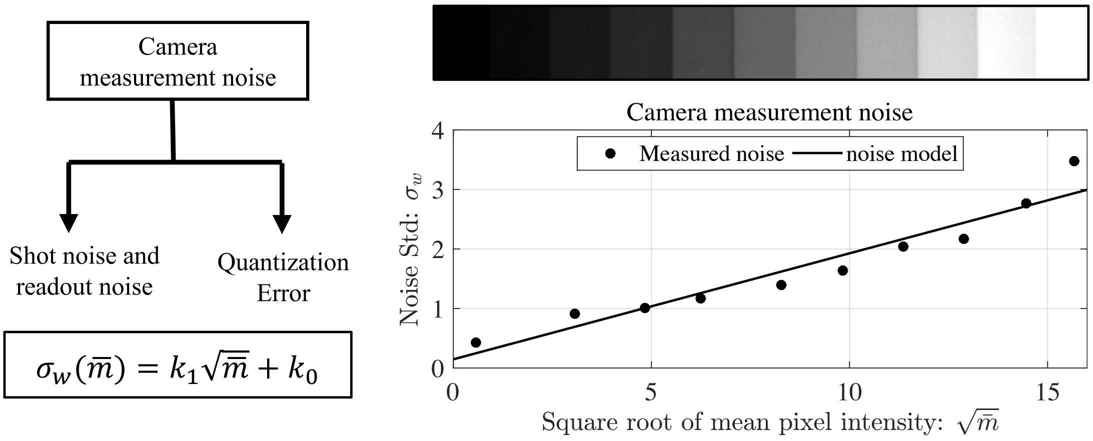

Figure S5: **Camera Measurement noise model and its validation:** The camera's measurement noise is dominated by photon shot noise, readout noise and quantization error, and the standard deviation (of the noise) can be modeled as $\sigma_n = k_1\sqrt{\bar{m}} + k_0$. Grey-scale chart is used to measure the standard deviation of the measurement noise as a function of the mean pixel intensity, and we fit the noise model to determine $K_0 = 0.146$ and $K_1 = 0.18$.

In absence of any other source of error, e.g. motion artifact, the standard deviation of the perfusion estimate obtained using the ML-estimator $\sigma(\hat{A}_{\text{ML}})$ (Equation 6) depends solely on the standard deviation of camera's measurement noise, i.e.

$$\sigma(\hat{A}(\vec{x})_{\text{ML}}) = \frac{\sigma_n(\bar{m})}{\|\mathbf{p}(t)\|} = \frac{B}{(2K+1)}\frac{\sigma_w(\bar{m})}{\bar{m}\|\mathbf{p}(t)\|} = \frac{B}{(2K+1)}\frac{(K_1\sqrt{\bar{m}} + K_0)}{\bar{m}\|\mathbf{p}(t)\|} \quad \text{(S4)}$$

where B is the normalized bandwidth of the bandpass filter used while computing the "AC over DC" ratio ($B = 0.62$), $(2K + 1)$ is the spatial averaging block size, $\|\mathbf{p}(t)\|$ is the L^2 norm of the reference pulse signal over $2T + 1$ samples, $\bar{m}$ is the mean intensity in the skin region, and K_0, K_1 are the measured constants for our camera's measurement noise model ($K_0 = 0.146, K_1 = 0.178$). The standard deviation of PulseCam's perfusion estimate $\sigma(\hat{A}_{\text{Robust}})$ (Equation 8) based on the robust M-estimator will be slightly higher than the standard deviation of the ML-estimator. We tuned the parameter of the Tukey bi-square loss function used to have 95% relative efficiency such that $\sigma(\hat{A}_{\text{PulseCam}}) = \sigma(\hat{A}_{\text{ML}})/0.975$. For quantitative comparison, here we define expected percent error in perfusion estimate as the ratio $\sigma(\hat{A}_{\text{Robust}})/E(\hat{A}_{\text{Robust}})$.

For the analysis that follows, we utilized the first one minute video recording (during no occlusion) from the arterial and venous occlusion dataset. Specifically, we used the palm video from participants three participants that represents the dark (Fitzpatrick type V or VI), olive and (Fitzpatrick type III or IV) and fair (Fitzpatrick type I or II) skin tone types. Figure S6 shows how the expected percent error in perfusion estimate varies for participants of the three skin tones (dark, olive, fair) as we changed (a) spatial averaging parameter $(2K + 1)$, (b) temporal averaging parameter $(2T + 1)$, and (c) average light intensity parameter $(\bar{m})$ in Equation (S4). Clearly, the choice of $(2K + 1)$ and $(2T + 1)$ provides a trade-off between the spatio-temporal resolution and the expected percent error in perfusion estimate due to measurement noise. Further, increasing the light intensity reduce the expected percent error in perfusion estimate. But, we cannot increase the light intensity beyond a certain level as it leads to pixel saturation, i.e. $\bar{m}$ at few locations on the skin surface may saturate to the maximum value of 256. Based on these results, we chose $(2K + 1) = 9$ and $(2T + 1)/\text{Fs} = 10$ (sec) (Fs is camera's sampling rate) for Fitzpatrick skin type I to IV participants and $(2K + 1) = 9$ and $(2T + 1)/\text{Fs} = 20$ for Fitzpatrick skin type V to VI participants to have sufficient spatio-temporal resolution with

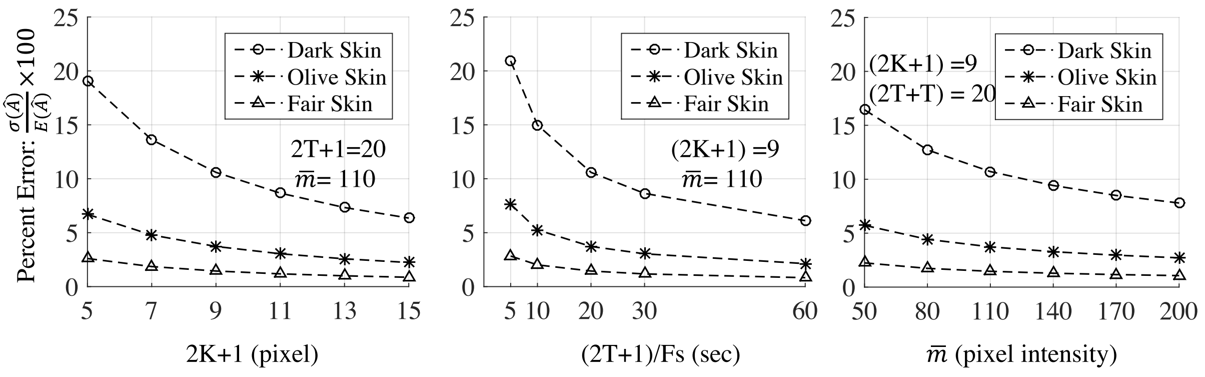

(a) Spatial averaging (b) Temporal averaging (c) Light Intensity

Figure S6: **Impact of spatio-temporal averaging and light intensity on percent error in perfusion estimate**: Figure shows how the percent error in perfusion estimate can be reduced by (a) increasing the spatial averaging $(2K+1)$, (b) increasing the temporal averaging $(2T+1)$ and (c) increasing the average light intensity $\bar{m}$.

percent perfusion error due to measurement noise to be below 10% for all skin tones.

S.5 Arterial and venous occlusion study

S.5.1 Experimental Setup

All the video recordings during the experiment are performed using a Point Grey FL3-U3-13E4C-C color camera [7]. We used two white diffused LED light source (PowerSmith PWL1130BS 30W) placed adjacent to the camera and approximately 1 m away from the imaged body part to achieve diffused and uniform illumination as suggested in [1] to minimize Ballistocardigram-related motion artifacts. To reduce specular reflection, we placed an H-polarized linear polarizer in front of the LED light and a V-polarized linear polarizer in front of the camera's objective. This perpendicular alignment of polarizers helps reject specular reflection from the skin surface and only allows scattered light from the subsurface to enter the camera. We minimized any other non-polarized ambient light to fall on the skin surface. All video recordings were performed at 30 frames per second (fps). The exposure is set to 8 msec, and the analog gain is set to 1.0 (i.e., 0 dB) thereby utilizing the full well capacity of the CMOS sensor.

Figure 2(a) depicts our experimental setup for the arterial and venous occlusion experiment. The participants were instructed to sit comfortably, and their left hand was placed on a support to minimize unintentional motion. A photoplethysmography sensor, i.e., the reference PulseOx (BIOPAC's TDS200 [8]) is positioned at the index finger of the right hand to obtain reliable reference pulse waveform (Note: Blood flow to the right hand is not occluded during this study). Another PulseOx (BIOPAC's TDS200 [8]) was attached to the index finger of the left hand (occlusion hand) to obtain contact-based perfusion index measurements derived from the PPG waveform during the occlusion experiment. The camera recording and the reference photoplethysmography sensors are started together using an automation script running on the acquisition PC. A manual pressure cuff is placed on the upper left arm of the participant to occlude either the artery or the veins by applying an appropriate external pressure.

ID	Gender (M/F)	Blood pressure (Sys / Dias)	Full occlusion pressure (mmHg)	Partial occlusion pressure (mmHg)	Fitzpatrick Skin type
1	M	$110/75$	140	$80-85$	IV
2	F	$98/57$	140	$60-65$	V or VI
3	M	$95/50$	115	$50-60$	I or II
4	M	$108/66$	130	$60-70$	V or VI
5	F	$117/75$	140	$70-80$	I or II
6	M	$114/64$	135	$60-70$	V or VI
7	M	$120/67$	140	$70-80$	I or II
8	F	$110/60$	130	$60-65$	III
9	F	$80/57$	100	$60-65$	III
10	M	$110/65$	130	$65-70$	IV
11	M	$110/65$	130	$65-70$	IV
12	F	$90/55$	110	$60-65$	V or VI

Table S1: Summary of the pressure-cuff settings and Fitzpatrick skin type for the 12 participants during the arterial and venous occlusion experiment.

S.5.2 Experimental procedure

The occlusion dataset consists of both venous (i.e. partial) occlusion as well as arterial (i.e. full) occlusion experiment which is done on 12 healthy participants (5 female, 7 male; aged $22-30$ years) of varying skin tones (Fitzpatrick skin type I to VI). The investigation protocol is approved by Rice's IRB (Protocol No. $928192-1$) and informed consent is taken from all participants. The steps followed during this experiment are:

(i) Arterial occlusion dataset: We first record the video of the palm under normal flow (without any occlusion) for a duration of 1 min. Then, we apply a pressure of around 20 mmHg above the systolic pressure (of the participant) to completely occlude the flow of blood to the palm for a duration of 1 min. We then release the occlusion and record an additional 2 mins of palm video.

(ii) Venous occlusion dataset: We first record the video of the palm under normal blood flow for a duration of 1 min. Then we applied a pressure $5-10$ mm Hg above the diastolic pressure (of the participant) for a duration of 2 min to completely stop the venous back-flow and only allow arterial inflow to the palm. We then release the occlusion and record additional 2 minute video of the palm.

We summarize the specific settings of the pressure cuff and the skin tone type of each participant in Table S1. Based on the Fitzpatrick scale [9], we categorize the 12 participants into four skin tone groups: (i) Fitzpatrick Skin Type I and II (Ivory, fair or pale), (ii) Fitzpatrick Skin Type III (fair with golden undertone), (iii) Fitzpatrick Skin Type IV (Olive or light brown), and (iv) Fitzpatrick Skin Type V and VI (dark brown and black).

S.5.3 Data analysis

We used the PulseCam algorithm as discussed in the methods section to obtain the spatio-temporal blood perfusion estimate $\hat{A}(\vec{x},t)$. We set the window size parameter to be 10 sec with a 9 sec overlap for Fitzpatrick skin type I to IV. For Fitzpatrick skin type V and VI, we set window to be 20 sec and a 19 sec overlap. The spatial averaging parameter $(2K+1)=9$ for all skin types. Therefore, we obtain spatial maps of perfusion at 1 sec time interval.

S.6 Incremental vascular occlusion study

To quantify the sensitivity of PulseCam, i.e. to measure how small a change in blood perfusion can be detected, we employ incremental vascular occlusion experiment [10]. In this experiment, we incrementally occluded the blood flow to the palm using a manual pressure cuff put at the upper arm. We videotape the palm, and used PulseCam to obtain blood perfusion estimate in different regions of the palm at different blood occlusion pressure, e.g., at 10 mmHg, 20 mmHg, etc. We analyzed the blood perfusion estimate to determine the sensitivity of PulseCam in detecting small changes in blood perfusion associated with incremental occlusion.

S.6.1 Spatial perfusion ratio

Blood perfusion in any body part, e.g. the palm in this case, may vary due to multiple endogenous and exogenous factors. Usually,expansion and contraction of the smooth muscle cause local variations in blood flow. These local variations happen due to changing metabolic demands of the tissue and is usually mediated by the sympathetic (vascular) tone. Similarly, the body reacts to external temperature to preserve or dissipate heat from the body by decreasing or increasing the peripheral blood perfusion. Also, breathing can also influence local blood flow. Obviously, many of these factors can not be controlled experimentally. Therefore, these exogenous and endogenous factors has the potential to act as confounding factors that can cause unintended changes in tissue perfusion, and can interfere with our intended controlled occlusion experiment. We had neglected these factors of variations during the arterial and venous occlusion experiment as the induced blood flow variations are usually very large. However, in the incremental vascular occlusion study, the intent is to induce much smaller changes in tissue perfusion to measure the sensitivity of PulseCam. Therefore, we cannot neglect the confounding factors anymore.

To better understand the impact of the confounding factors, we conducted a preliminary experiments where we videotaped both the palms of a participant for 5 min without inducing any blood flow occlusion and obtained blood perfusion estimate using PulseCam. Figure S7 shows the temporal trend of perfusion from symmetric region-of-interest (ROIs) in both the palms (left and right as shown). The coefficient of variability (COV), i.e. the ratio of the standard deviation to the mean of the temporal trend, of individual perfusion estimate in the selected ROI in the left and right palm are 0.383 and 0.294 respectively over the 5 min video recording. The relatively high COV clearly indicates that these confounding factors of blood perfusion variations cannot be neglected during incremental vascular occlusion experiment. However, the temporal trend of perfusion from symmetric ROIs in both the palms (left and right) are highly correlated with $R = 0.97$. We found similarly high correlation between perfusion obtained from other symmetric ROIs in the palm and for other participants. This clearly indicates that the confounding factors impact the peripheral perfusion in symmetric regions of the palm in a similar way Therefore, we propose to cancel out these confounding factor of blood perfusion variations by computing a ratio of perfusion estimate at each time instant between anatomically symmetric parts of the left and the right palm. We will call such a ratio as *spatial perfusion ratio*. The COV for the computed spatial perfusion ratio during the preliminary experiment reduces to 0.14 indicating, at least, partial cancellation of the confounding factors.

We want to clarify that the proposed empirical approach of computing the spatial ratio of perfusion to cancel out common factors of blood flow variations assumes that the metabolic demand in both the palms are similar and do not change significantly during the coarse of the experiment and that the participants do not have any medical condition that might significantly alter blood flow in one of their palm in relation to the other. With these assumptions in mind, in

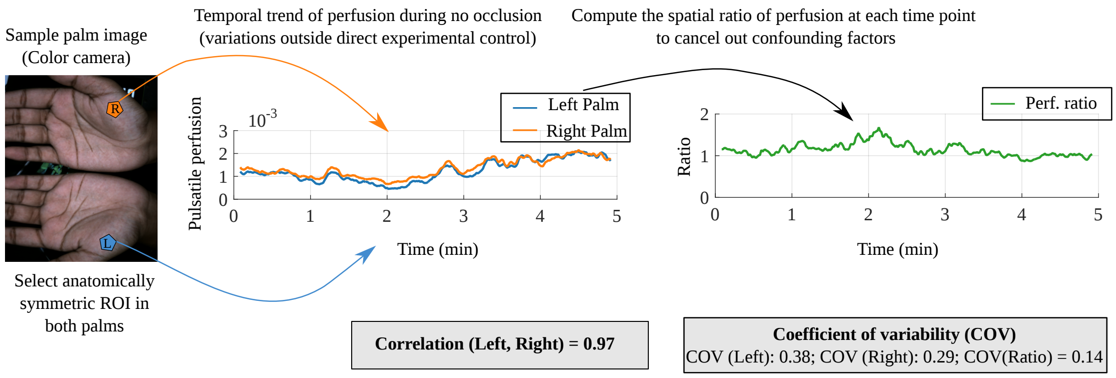

Figure S7: **Spatial perfusion ratio**: Peripheral perfusion in the palm can change due to physiological factors outside direct experimental control. We found that the perfusion variations in symmetric regions of both the palm are highly correlated. So, computing spatial perfusion ratio can potentially cancel out these confounding factors to a large extent as is evident from the lower coefficient of variation for the ratio..

the incremental vascular occlusion experiment, we ask the participants to sit comfortably and keep both the palm at rest for at least 3 min before the start of the data recording to ensure that the metabolic demands of the two palm stabilizes and are similar. Also, we only recruit healthy adults who do not have any known medical conditions that might impair blood flow in one of their palms.

S.6.2 Experimental setup

In this study, we record the video of both the palms at 30 FPS a color Point Grey FL3-U3-13E4C-C camera [7] as shown in Figure 2(e). We used two white diffused LED light source (PowerSmith PWL1130BS 30W) placed adjacent to the color camera and approximately 1.5 ft away from the imaged palm to achieve diffused and uniform illumination of the palm. The LED light source and the camera are placed on top of a Acrylic box facing downwards to image the palm. To reduce specular reflection from the skin surface, we put an H-polarized linear polarizer in front of the LED light and a V-polarized linear polarizer in front of the color camera's objective. For comparisons with contact-based perfusion index measurements (i.e. PI), we placed MightySat Fingertip PulseOx from Masimo [11] on the middle finger of each palm (O-Palm and R-Palm). We also place a Contec CMS50D+ Finger pulse Oximeter on the R-Palm's index finger to record reference pulse (photoplethysmogram) waveform which is required to estimate blood perfusion maps in both the palms using the PulseCammethodology [12]. All the data streams are recorded synchronously using a custom written software running on a Laptop computer.

S.6.3 Experimental procedure

We first measured the diastolic and systolic blood pressure of each participant. Then, the participants were asked to rest both the palms on a cushioned surface inside the clear acrylic box facing the plantar side of the palms upwards. For each participant, we incrementally occluded the blood flow to the O-palm using a pressure cuff put at the upper arm. We started with a 10 mmHg

occlusion pressure, and then increase the occlusion pressure in increments of 10 mmHg, i.e. at 20 mmHg, 30 mmHg ... up to 20 mmHg higher than the Systolic pressure of the participant. Each occlusion pressure was applied for a duration of one minute and is always followed by one minute of release time, i.e. no occlusion. Therefore, for a participant having 120 mmHg systolic pressure, the incremental occlusion protocol we followed is: RELEASE (1 min) - 10 mmHg OCCLUSION (1 min) - RELEASE (1 min) - 20 mmHg OCCLUSION (1 min) ... - RELEASE (1 min) - 140 mmHg OCCLUSION (1 min) - RELEASE (1 min) for a total of 29 min of video recording. The experimental procedure is pictorially depicted in Figure 2(f). The investigation protocol is approved by Rice's IRB (Protocol No. $928192-1$) and informed consent is taken from all participants. We performed this experiment on 15 healthy adults (Male: 9, Female: 6) of diverse skin tones (Fitzpatrick skin type I to V) in the $24-36$ years age group.

S.6.4 Data analysis

From the palm videos recorded using the color camera, we obtain blood perfusion maps $a_{\mathrm{G}}(\vec{x}, t)$. Here, 'G' stands for the green channel of the color camera (we only used green channel in this study), $\vec{x}$ denote the spatial location on the palm, and t denotes the time. We set the parameters in the PulseCam algorithm to produce blood perfusion index estimates at 1 sec time interval. We also obtain contact-based perfusion index measurement $M_O(t)$ and $M_R(t)$ from the MightySat PulseOx put on the middle finger of the O-palm and the R-palm respectively at every 2 sec interval.

We can estimate local blood perfusion index at any region-of-interest (ROI) within the palm by averaging the obtained camera-based blood perfusion map within that ROI, i.e.

$$A_{\mathcal{X},\lambda}(t) = \frac{1}{|\mathcal{X}|} \sum_{x \in \mathcal{X}} (a_{\lambda}(\vec{x}, t)) \quad \lambda \in \{\mathrm{G}, \mathrm{NIR}\}, \tag{S5}$$

where $\mathcal{X}$ denote the set of pixels inside the selected ROI.

To reduce the impact of confounding variations during our incremental occlusion experiment, we computed a spatial ratio of perfusion estimates at anatomically symmetric ROIs in both the palms, i.e. we compute a ratio of perfusion estimate

$$R_{\mathcal{X},\lambda}(t) = \frac{A_{\mathcal{X}_O,\lambda}(t)}{A_{\mathcal{X}_R,\lambda}(t)} \quad \text{where } \mathcal{X}_O, \mathcal{X}_R \text{ are symmetric ROI in both palms.} \tag{S6}$$

For this study, we manually select symmetric ROIs in the R-palm and the O-palm. We similarly computed the ratio of contact-based perfusion index estimates measured using the two MightySat PulseOxfor fair comparison, i.e. $S(t) = \frac{M_O(t)}{M_R(t)}$.

To get an estimate of blood perfusion at each occlusion pressure level, we averaged the computed perfusion ratio over a duration of 30 sec in between each occlusion and release events, i.e.

$$R^{0_1}_{\mathcal{X},\lambda} = \frac{1}{30} \sum_{t=15}^{45} R_{\mathcal{X},\lambda}(t), \; R^{10}_{\mathcal{X},\lambda} = \frac{1}{30} \sum_{t=75}^{105} R_{\mathcal{X},\lambda}(t),$$
$$R^{0_2}_{\mathcal{X},\lambda} = \frac{1}{30} \sum_{t=135}^{165} R_{\mathcal{X},\lambda}(t), \; R^{20}_{\mathcal{X},\lambda} = \frac{1}{30} \sum_{t=195}^{225} R_{\mathcal{X},\lambda}(t) \; \ldots, \tag{S7}$$

where the superscript denote the corresponding pressure value in mmHg, and there are multiple instances of 0 mmHg as each occlusion pressure is preceded by one minute of no occlusion in the experimental protocol. Similarly, we can obtain contact-based perfusion ratio as measured using the MightySat PulseOx for each occlusion pressure, e.g. $S^{0_1}, S^{10}, S^{0_2}, S^{20}, \ldots$ etc.

S.7 Perfusion monitoring during surgery

S.7.1 Experimental setup

We recorded the video of patient's palm during the surgery using a Point Grey FL3-U3-13E4C-C color camera [7] placed approximately 8 inches away from the palm. Due to space constraints in the surgery room, we could only use one LED light source (PowerSmith PWL1130BS 20W) which was placed adjacent to the camera and approximately 1 foot away from the patient's palm. We placed polarizers both on the camera as well as on the light source to minimize specular reflection. However, we could not restrict unpolarized ambient light to fall on the palm as we did not have any control on the operating room lights. We used tripods to place the camera and the light source at appropriate positions near the patient. Also, we placed two PulseOx on patient's finger: (i) A CMS 50D+ PulseOx to record the reference pulse waveform required by the PulseCam methodology, and (ii) a Masimo MightySat PulseOx to record the contact-based perfusion index (PI) for comparison. All the data (palm video and PulseOx measurements) were recorded synchronously using a custom-built software running on a data acquisition laptop. The data from the Masimo MightySat PulseOx is synced to a smart phone over Bluetooth. The anesthesia induction was done using fentanyl, propofol, rocuronium and then sevofluorane. We also recorded the exact time when the anesthesia was inducted and the time of the onset of anesthesia as well as patient's systolic and diastolic blood pressure several times during the experiment.

S.7.2 Data analysis

We used the PulseCam methodology to combine the video recording and the reference pulse waveform to obtain spatio-temporal blood perfusion estimate $\hat{A}(\vec{x}, t)$. For all the videos in the anesthesia monitoring study, we set analysis window size parameter to equal 20 sec with an overlap of 19 sec. Thus, we obtained blood perfusion map estimate at 1 sec time interval. The Masimo's MightySat PulseOx provided perfusion index measurement at 2 sec time interval.

S.7.3 Data rejection

Out of the 10 patients data we recorded, we had to reject two patient data, One patient data (Patient ID: 7) was rejected as no anesthesia was inducted during the surgery. Another patient data (Patient ID: 2) was rejected due to corrupted reference blood volume recording because of a loss of contact of the PulseOx with patient's skin. Here, we analyzed the remaining 8 patients data. Also, for another patient (Patient ID: 5), there was an automatic pressure cuff placed around the arm of the patient unintentionally during the surgery which intermittently turned on and off causing significant variations in the measured pulsatile blood perfusion in the palm. As the timing of these pressure measurements did not overlap with anesthesia induction and onset times, so we kept the patient data in the analysis set.

For few patient's data, we have to reject initial $1 - 2$ min video recording as the palm was occluded from camera's view. This usually happened because the data collection personnel performed few adjustment to the PulseOx, the camera or the light source after starting the data recording. In Table S2, we have summarized the analysis start time, analysis end time, anesthesia induction time, anesthesia onset time, and data analysis duration for each of the remaining 8 patients we analyzed. All timings are relative to the time of pressing the START button in the custom built recording software running on the laptop.

PID	Analysis start time (m:ss)	Analysis end time (m:ss)	Anesthesia induction (m:ss)	Anesthesia onset time (m:ss)	Duration (sec)
1	0 : 45	6 : 15	1 : 11	2 : 00	330
3	0 : 00	5 : 30	1 : 50	4 : 53	330
4	1 : 30	9 : 00	3 : 32	5 : 20	450
5	1 : 30	8 : 30	1 : 51	4 : 43	420
6	0 : 00	8 : 30	3 : 03	4 : 02	510
8	2 : 00	9 : 00	2 : 46	4 : 13	420
9	4 : 00	24 : 00	11 : 15	15 : 05	1200
10	0 : 00	14 : 30	7 : 56	10 : 09	870

Table S2: A summary of the analysis start time, analysis end time, anesthesia induction time, anesthesia onset time, and analysis duration for the 8 (out of 10) patients in surgery room study. We did not analyze PID 7 as anesthesia was not induced and PID 2 as the reference pulse waveform was severely corrupted.

S.7.4 Reference pulse validity test

Due to the ongoing activities related to the surgery, it is usually difficult to acquire error-free reference pulse waveform using the contact-based PulseOx in the operating room. The reference waveform usually gets corrupted due to either loss of contact with the skin surface or due to motion artifact. As our proposed PulseCam methodology assumes an error-free reference pulse waveform, any error in the reference pulse waveform would lead to corrupted blood perfusion map estimate.

To address this issue, we devise a simple signal validity test that checks the correctness of the reference pulse waveform recorded using the contact-based PulseOx. We apply two tests to determine the validity of the recorded pulse waveform: (i) the saturation test determines if the PulseOx signal is saturated due to lack of contact with the skin surface or due to motion artifact; and (ii) the goodness test that computes a surrogate measure of signal-to-noise ratio to reject motion corrupted samples from the recorded pulse waveform.

Saturation test: To test for sensor saturation, we first compute the derivative of the recorded reference PulseOx signal $p_{\text{ox}}(t)$ and check if the derivative is zero for a contiguous S or more samples. Thus, using this saturation test, we can reject corrupted pulse waveform samples due to loss of contact with the skin surface or due to sudden and large movement. We set the $S = 20$ to reject pulse waveform samples that are saturated for continuous 0.33 sec as the sampling rate of the reference PulseOx is 60 Hz.

Goodness test: The pulse waveform is a nearly periodic signal, and therefore all the signal power in the spectral domain is concentrated around the pulse rate. Motion artifacts adds noise to the signal and usually the power of the added noise is distributed everywhere in the pass-band, i.e. between 0.5 Hz to 5 Hz. Thus, a surrogate measure of the signal to noise (power) ratio can be defined as the ratio of the power of the recorded waveform around the pulse rate to the power everywhere else. We will call this ratio as the goodness metric as it is based on our past work on camera-based pulse estimation [13].

$$G(t) = \frac{\int_{\text{PR}-b}^{\text{PR}+b} P_{\text{ox}}(f,t)df}{\int_{0.5}^{5} P_{\text{ox}}(f,t)df}, \tag{S8}$$

where $P_{\text{ox}}(f,t)$ is the spectrogram of the recorded pulse waveform computed using the short-time Fourier transform. We consider the pulse waveform to be free from motion artifact if the

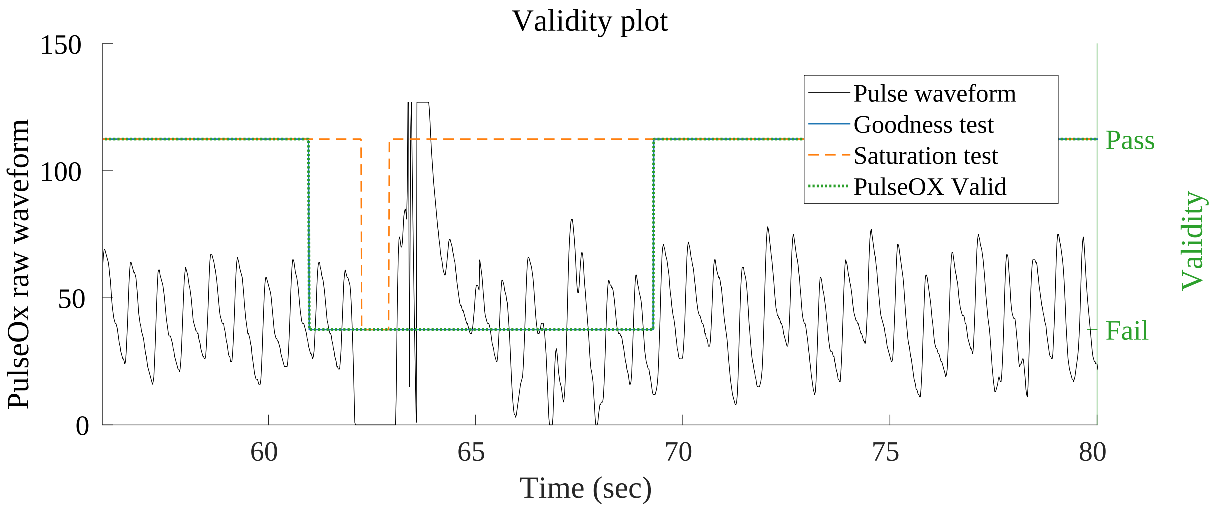

Figure S8: **Reference pulse validity test**: In clinical settings, the reference PulseOx may be corrupted due to either loss of contact with the skin surface or motion artifact. A pulse waveform validity test can be performed by combining a saturation test and a Goodness test to obtain a pulse validity signal. If the pulse validity fails, then we do not consider corresponding video frames for further analysis by the PulseCam algorithm.

goodness metric is above 5.

Finally, we combined the saturation test condition and the goodness test condition to obtain a signal validity test for the reference pulse waveform recorded using the PulseOx. Figure S8 shows a sample pulse waveform recorded during surgery for a patient and the corresponding saturation test, goodness test and pulse validity test results.

We slightly modified the PulseCam to take both the reference pulse signal as well as the pulse validity signal as input to the algorithm. If the pulse validity is FALSE, then we do not consider the corresponding video frames for computing the blood perfusion map estimate during the analysis window. Even if we reject few frames, we can still compute a reliable perfusion estimate as the analysis window size is chosen to be 20 sec. However, if the pulse signal is corrupted for a combined duration of 10 sec or more within any 20 sec analysis window, then we do not compute the perfusion estimate altogether and report NA as the output of the PulseCam algorithm.

S.7.5 Spatial map and temporal trend of blood perfusion

Figure S9 shows the spatial blood perfusion map in the palm region for the 8 patients at three time instants: (i) just before anesthesia induction (left), 90 secs after anesthesia induction (center), and at the time of the onset of anesthesia (right) as determined by the expert anesthesiologist. These spatial maps of blood perfusion clearly highlights that there is a distinct increase in peripheral perfusion due to vasodilation just after anesthesia induction, and the pulsatile perfusion tends to remain high throughout the anesthesia onset. The increase in peripheral perfusion is specially large in the radial and the ulnar regions of the palm as can be seen from these spatial maps.

For further analysis, we consider four distinct regions in the palm based on the arteries that supply blood to the region: (i) radial region, (ii) ulnar region, (iii) palmar arch region, and (iv) central regions. Since in this pilot study, we are not interested in inter-subject variability in peripheral perfusion, so we also normalized the spatio-temporal blood perfusion estimate by the mean perfusion in the palm region during the first 20 sec of the data recording for each

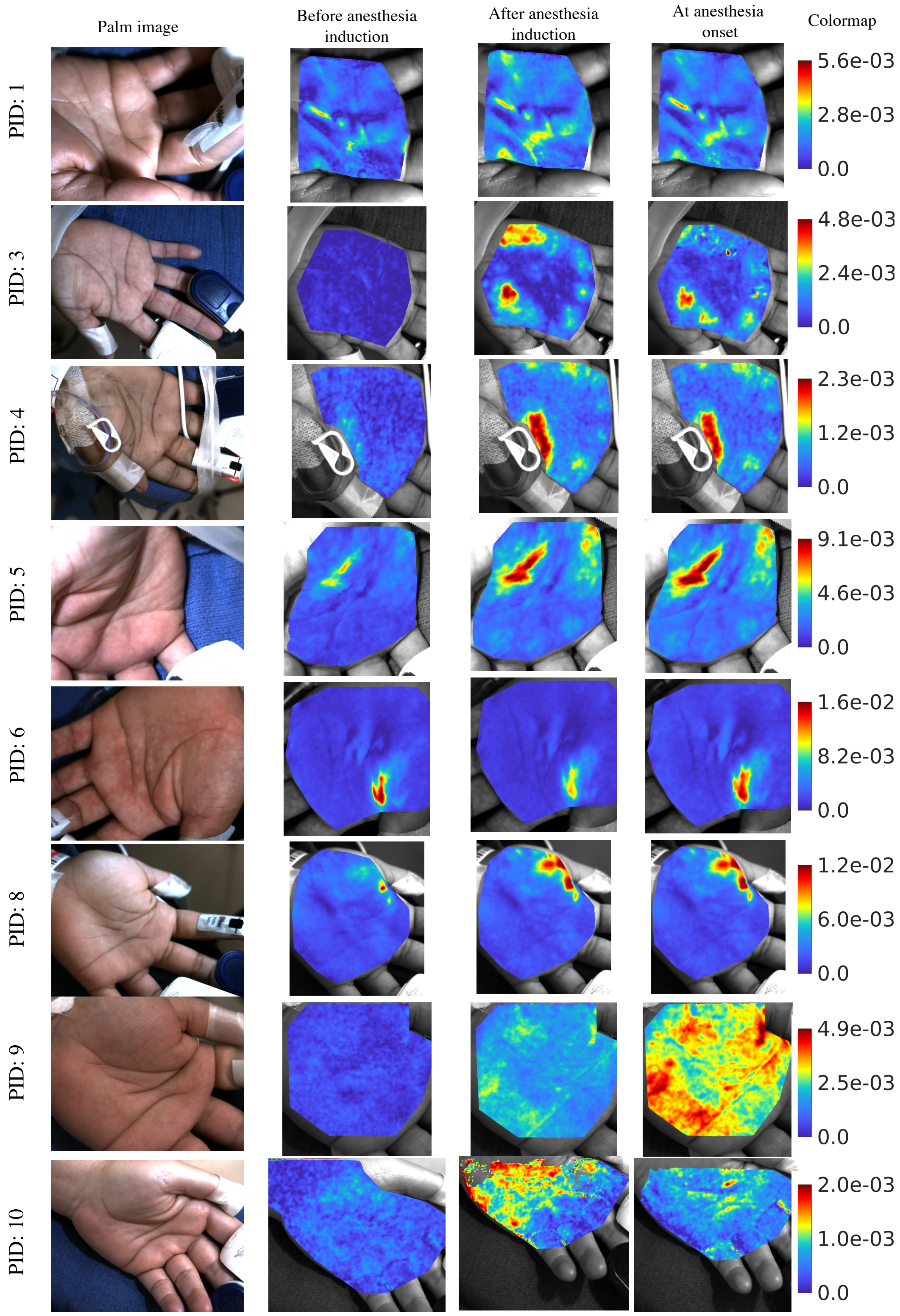

Figure S9: **Spatial blood perfusion map at different stages of anesthesia induction**

participant to obtain normalized blood perfusion estimate. We followed similar normalization steps for the perfusion index measurement obtained using the Masimo's MightySat PulseOx.

Figure S10 shows the temporal variations in peripheral perfusion in the four selected spatial regions of the palm as well as the perfusion index (PI) measurement obtained from the Masimo MightySat PulseOx attached to the patient's finger (shown in black) for all the participants. The specific time at which anesthesia is inducted is also marked. These spatio-temporal trend in peripheral perfusion clearly indicates sudden change in the dynamics of peripheral perfusion just after the anesthesia induction for all but one patient (PID: 6).

For one specific patient (PID: 6), neither the PulseCam nor the PulseOx detected any change in peripheral perfusion before and after anesthesia induction. This was unusual as all the other 7 patients show small or large increase in PulseCam derived peripheral perfusion estimate post anesthesia induction. One potential reason for this could be the unusually low systolic blood pressure (hypotention) of only 73 mmHg at the time of anesthesia induction and only 87 mmHg at the time when anesthesia sets in. Usually, at such low systolic BP there is hardly any pulse pressure at the wrist, e.g. the radial pulse, and vasodilation caused by anesthesia induction would lead to imperceptible change in peripheral perfusion.

S.8 Supplemental videos

S.8.1 Arterial and venous occlusion

Video URL: `https://goo.gl/7TqFdc`
Summary: The video shows the blood perfusion map in the palm of a participant while we occlude the arterial blood flow using a manual pressure cuff at 140 mmHg. PulseCam can faithfully recover reduction in pulsatile blood flow in the palm during occlusion and a sudden increase in blood flow after release.

S.8.2 Incremental vascular occlusion

Video URL: `https://goo.gl/iJmh1h`
Summary: The video shows the blood perfusion map in both the palms while we occlude the the flow of blood to one of the palm (left palm). We use manual pressure cuff to incrementally occlude the blood flow with 10 mmHg, 20 mmHg . . . 100 mmHg occlusion pressure. PulseCam can easily detect blood perfusion changes due to even 10-20 mmHg occlusion pressure difference that is difficult to identify using existing modalities.

S.8.3 Perfusion monitoring during surgery

Video URL: `https://goo.gl/qGpPMz`
Summary: The video shows the blood perfusion map in the palm of a patient undergoing surgery in which general anesthesia is used. Anesthesia induction is known to cause an increase in peripheral blood perfusion. PulseCam can easily detect an increase in blood perfusion in the palm just after anesthesia induction. Also, PulseCam can recover these perfusion changes in a challenging clinical setting.

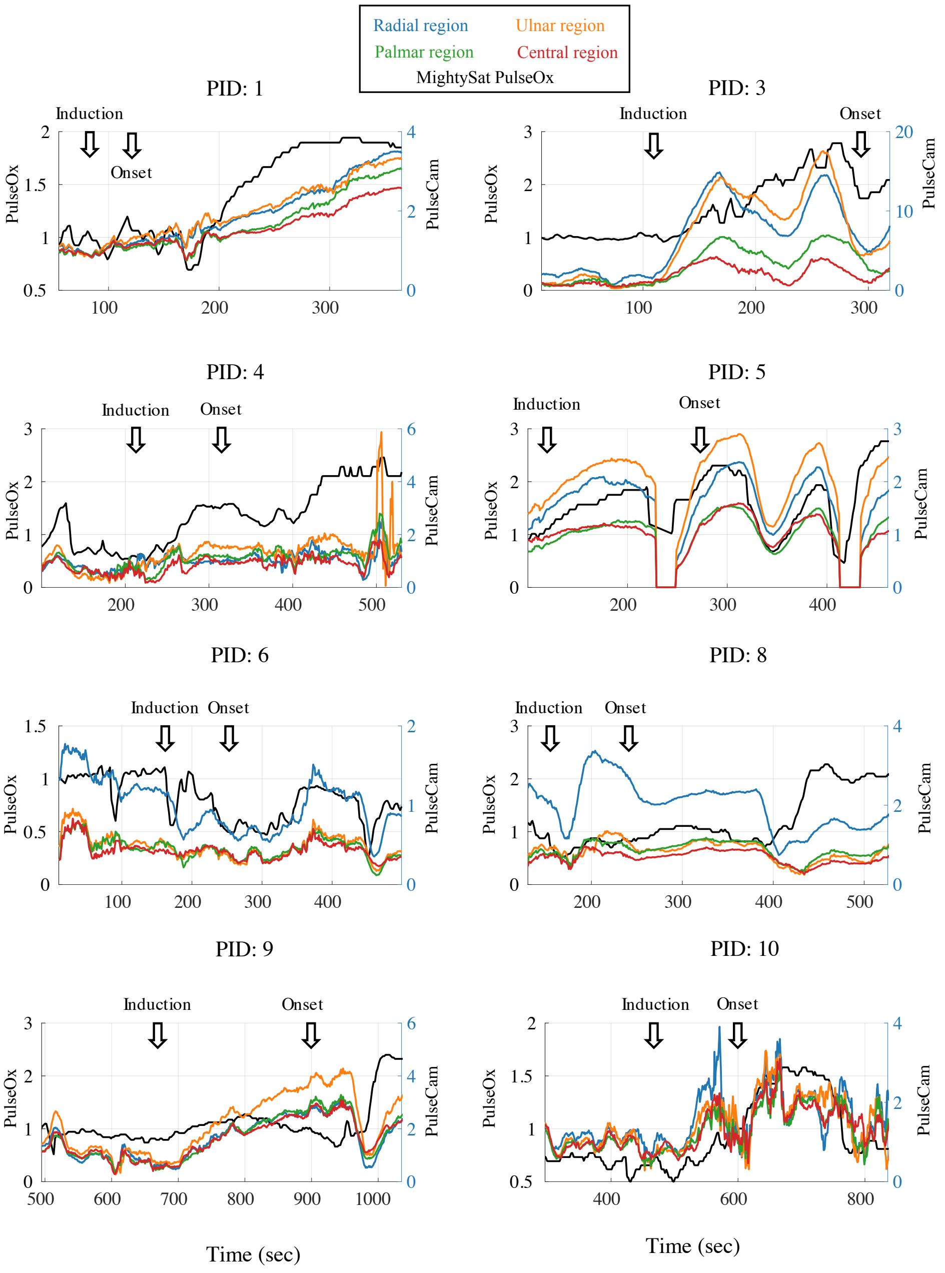

Figure S10: Temporal trend of peripheral perfusion before, during and after anesthesia induction for 8 out of the 10 patients in the study. The black plot shows perfusion estimate using a PulseOx whereas the colored plots show perfusion estimate using PulseCam in different regions of the palm marked in Figure 4(c)).

References

[1] Moco, A., Stuijk, S. & de Haan, G. Ballistocardiographic artifacts in PPG imaging. *IEEE Transactions on Biomedical Engineering* 1 (2016). URL http://ieeexplore.ieee.org/lpdocs/epic03/wrapper.htm?arnumber=7332770.

[2] Trumpp, A., Bauer, P. L., Rasche, S., Malberg, H. & Zaunseder, S. The value of polarization in camera-based photoplethysmography. *Biomedical Optics Express* **8**, 2822 (2017). URL https://www.osapublishing.org/abstract.cfm?URI=boe-8-6-2822.

[3] Moço, A. V., Stuijk, S. & de Haan, G. Skin inhomogeneity as a source of error in remote PPG-imaging. *Biomedical Optics Express* **7**, 4718 (2016). URL https://www.osapublishing.org/abstract.cfm?URI=boe-7-11-4718.

[4] Zachiu, C., Papadakis, N., Ries, M., Moonen, C. & Denis de Senneville, B. An improved optical flow tracking technique for real-time MR-guided beam therapies in moving organs. *Physics in Medicine and Biology* **60**, 9003–9029 (2015). URL http://www.ncbi.nlm.nih.gov/pubmed/26540256http://stacks.iop.org/0031-9155/60/i=23/a=9003?key=crossref.8e9f5a4034d646a52689c66d47c1d7b1.

[5] Moço, A. V., Stuijk, S. & de Haan, G. Motion robust PPG-imaging through color channel mapping. *Biomedical optics express* **7**, 1737–1754 (2016). URL http://www.ncbi.nlm.nih.gov/pubmed/27231618{\%}7B{\%}25{\%}7D5Cnhttp://www.pubmedcentral.nih.gov/articlerender.fcgi?artid=PMC4871078.

[6] Bouguet, J.-y. & Bouguet, J.-y. Pyramidal implementation of the Lucas Kanade feature tracker. *INTEL CORPORATION, MICROPROCESSOR RESEARCH LABS* (2000). URL http://citeseerx.ist.psu.edu/viewdoc/summary?doi=10.1.1.185.585.

[7] Flea3 1.3 mp color usb3 vision (e2v ev76c560). https://www.ptgrey.com/flea3-13-mp-color-usb3-vision-e2v-ev76c560-camera. Accessed: 2018-04-16.

[8] Biopac mp150 starter system). https://www.biopac.com/product-category/research/systems/mp150-starter-systems/. Accessed: 2018-04-16.

[9] Fitzpatrick skin type. https://www.arpansa.gov.au/sites/g/files/net3086/f/legacy/pubs/RadiationProtection/FitzpatrickSkinType.pdf. Accessed: 2018-04-16.

[10] Abay, T. Y. & Kyriacou, P. A. Comparison of NIRS, laser Doppler flowmetry, photoplethysmography, and pulse oximetry during vascular occlusion challenges. *Physiological Measurement* **37**, 503–514 (2016). URL http://www.ncbi.nlm.nih.gov/pubmed/26963349http://stacks.iop.org/0967-3334/37/i=4/a=503?key=crossref.581ea9a50e3e3376ca7e4aa5affcf822.

[11] Mightysattm fingertip pulse oximeter. http://masimopersonalhealth.com/. Accessed: 2018-04-16.

[12] Kumar, M., Suliburk, J., Veeraraghavan, A. & Sabharwal, A. PulseCam: High-resolution blood perfusion imaging using a camera and a pulse oximeter. In *2016 38th Annual International Conference of the IEEE Engineering in Medicine and Biology Society (EMBC)*, 3904–3909 (IEEE, 2016). URL http://ieeexplore.ieee.org/document/7591581/.

[13] Kumar, M., Veeraraghavan, A. & Sabharwal, A. DistancePPG: Robust non-contact vital signs monitoring using a camera. *Biomedical Optics Express* **6**, 1565 (2015). URL https://www.osapublishing.org/boe/abstract.cfm?uri=boe-6-5-1565.
